# Vectorial folding of telomere overhang promotes higher accessibility

**DOI:** 10.1093/nar/gkac401

**Published:** 2022-06-10

**Authors:** Tapas Paul, Patricia L Opresko, Taekjip Ha, Sua Myong

**Affiliations:** Department of Biophysics, Johns Hopkins University, Baltimore, MD21218, USA; Department of Environmental and Occupational Health, University of Pittsburgh Graduate School of Public Health, and UPMC Hillman Cancer Center, Pittsburgh, PA15213, USA; Department of Biophysics, Johns Hopkins University, Baltimore, MD21218, USA; Physics Frontier Center (Center for Physics of Living Cells), University of Illinois, 1110 W. Green St., Urbana, IL 61801, USA; Howard Hughes Medical Institute, Johns Hopkins University, Baltimore, MD, USA; Department of Biophysics, Johns Hopkins University, Baltimore, MD21218, USA; Physics Frontier Center (Center for Physics of Living Cells), University of Illinois, 1110 W. Green St., Urbana, IL 61801, USA

## Abstract

Human telomere overhang composed of tandem repeats of TTAGGG folds into G-quadruplex (G4). Unlike in an experimental setting in the test tube in which the entire length is allowed to fold at once, inside the cell, the overhang is expected to fold as it is synthesized directionally (5′ to 3′) and released segmentally by a specialized enzyme, the telomerase. To mimic such vectorial G4 folding process, we employed a superhelicase, Rep-X which can unwind DNA to release the TTAGGG repeats in 5′ to 3′ direction. We demonstrate that the folded conformation achieved by the refolding of full sequence is significantly different from that of the vectorial folding for two to eight TTAGGG repeats. Strikingly, the vectorially folded state leads to a remarkably higher accessibility to complementary C-rich strand and the telomere binding protein POT1, reflecting a less stably folded state resulting from the vectorial folding. Importantly, our study points to an inherent difference between the co-polymerizing and post-polymerized folding of telomere overhang that can impact telomere architecture and downstream processes.

## Graphical Abstract

**Graphical Abstract:**
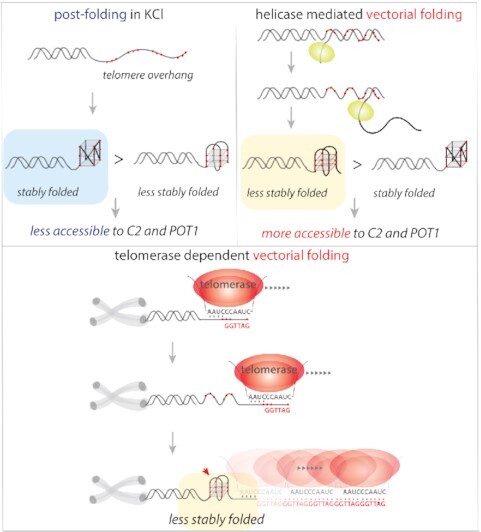
Vectorial folding of telomeric overhang leads to less stably folded G-quadruplex which yields higher accessibility to POT1.

## INTRODUCTION

Telomeres are specialized structures that cap the ends of linear chromosomes in eukaryotes (1). Human telomere DNA consists of several kilobases of double-stranded TTAGGG repeats followed by a 3′ single-stranded overhang of tandem TTAGGG repeats (∼50–300 nt) (2–4). Such G-rich overhang folds into G-quadruplex (G4) structures of different conformations mediated by the Hoogsteen-bonded G-quartet motifs which is stabilized by monovalent cations (5,6). The conformation and stability of G4 depend on the loop sequences and solution conditions (7–11). Previous studies using nuclear magnetic resonance (NMR), circular dichroism (CD), single-molecule fluorescence resonance energy transfer (smFRET) and X-ray crystallography have shown hybrid and parallel G4 conformations of human telomere overhang at physiological ionic condition (9,11–14). The presence of G4 at telomeres in the cell has been clearly demonstrated by using BG4 antibody which recognizes G4 with high affinity and specificity (15). In addition, a recent study directly confirmed the G4 folding of a nascent telomeric DNA while being extended by a telomerase (16).

Telomerase is a ribonucleoprotein complex that is uniquely built for synthesizing the TTAGGG repeats of the telomere overhang (17–19). The RNA component within a telomerase serves a dual function of directing the telomerase to the telomere and templating for TTAGGG repeat addition to the 3′ overhang (20). In most somatic cells, telomere DNA shortens due to the end replication problem, yet highly proliferative cells such as stem cells and tumor cells rely upon the telomerase activity to maintain the telomere length (21–23). Approximately 85% tumors use telomerase for telomere maintenance while ∼15% use the alternative lengthening of telomere (ALT) mechanism (24,25). Newly synthesized TTAGGG repeats is expected to fold into G4 structure anywhere along the G-rich strand (16,26). The exonuclease hydrolysis assay using (TTAGGG)_7_ suggested that the probability of G4 formation at the 3′ end is higher than the 5′ end (6,27). The folding mechanism of human telomeric G4 remains uncertain although different conformations have been suggested (11,28). Evidences suggest that G-hairpin and G-triplex form an intermediated during G4 folding (29). However, it remains uncertain what structures emerge from the newly extended telomere overhang.

Telomerase extends the telomere overhang by adding one GGTTAG repeat at a time from 5′ to 3′ direction and the newly synthesized overhang folds into G4 structure (16). Here, we sought to replicate such vectorial folding process by taking advantage of a processive helicase, Rep-X (30,31) as demonstrated in a previous study (27). We prepared a single-molecule platform where FRET labeled overhang of two to eight TTAGGG repeats in a duplex is unwound by Rep-X in 3′ to 5′ direction along the C-rich DNA strand, releasing the G-rich DNA in 5′ to 3′ direction and allowing it to fold into G4 (27). Our results reveal that the folded conformation achieved by the vectorial folding (vf) is significantly different from the conventional folding where the full sequence is allowed to fold at once (herein we refer to post-folding, pf). The same difference was observed when the rate of Rep-X unwinding matched that of the telomerase extension (∼1 nucleotide per second) by tuning the ATP concentration (50 μM) which slows down the Rep-X unwinding (32,33). Strikingly, the vectorially folded conformation leads to significantly higher accessibility towards complementary C-rich strand ([CCCTAA]2 i.e. C2) and telomeric single-stranded DNA binding protein POT1 (34,35). We also developed a 3′ to 5′ directed vectorial folding assay by using a λ-exonuclease which revealed a similar conformations and accessibility comparable to the Rep-X induced vectorial folding, suggesting that the impact of vectorial folding is independent of the directionality. The vectorial folding-specific accessibility increase is not simply due to an instantaneous folding of G4 in post-folding because LiCl to KCl buffer exchange does not recapitulate it. Taken together, our results reflect inherently different conformations that lead to higher accessibility of the nascently synthesized and folded telomeric G4 than the post-folded G4.

## MATERIALS AND METHODS

### Preparation of DNA constructs

The HPLC-purified DNA oligonucleotides (tabulated in Supplementary Table S1) containing both biotin for immobilization and Cy3, Cy5 for FRET measurements were purchased from IDT. The DNA constructs were designed in two different pathways. For post folding experiments: Each partial duplex DNA construct (10 μM) was prepared by mixing the biotin-conjugated DNA strand with its complementary strand at molar ratio of 1:1.2 (biotinylated:nonbiotinylated) and annealed in a buffer containing 10 mM Tris–HCl (pH 7.5) and 100 mM KCl. For vectorial folding experiments: Each partial duplex DNA construct (10 μM) was prepared by mixing the biotin-conjugated DNA strand with its complementary strand containing telomeric overhang and complementary sequence of telomere overhang with or without poly-thymine tail at molar ratio of 1:1.2:1.5 and annealed in a buffer containing 10 mM Tris–HCl (pH 7.5) and 5 mM MgCl_2_. All the DNA constructs were annealed in a thermocycler with the following program as reported earlier: 95°C for 2 min; gradual cooling to 40°C at the rate of 2°C/min; further cooling by 5°C/min until 4°C (35,36). The annealed constructs were stored at –20°C and freshly reannealed before use. Milli-Q water was used to prepare all buffers and then filtered through 0.22 μm membrane filters.

### Slide surface preparation

All smFRET measurements were carried out on polyethylene glycol (PEG) passivated quartz slides to avoid any non-specific surface interactions of excess DNA or protein. The slides and coverslips (Fisher Scientific, USA) were cleaned thoroughly with methanol, acetone and etched by 1 M potassium hydroxide. Then the slides were fully burned for two minutes and coverslips were quickly sterilized by passing through the flame 4–5 time to remove all source of fluorescence. Afterwards, the surfaces of both slides and coverslips were treated with aminosilane for 30–45 min and finally coated with a mixture of 98% mPEG (mPEG-5000, Laysan Bio, Inc.) and 2% biotin–PEG (biotin-PEG-5000, Laysan Bio, Inc) for 4–6 h. The slides and coverslips were then washed and dried using nitrogen gas and stored in −20°C for future experiments. For experimental purpose, the microfluidic sample chamber was created between the plasma cleaned slide and the coverslip was coated with PEG and biotin–PEG (37–40).

### Single-molecule FRET assays

Single-molecule FRET study was carried out with a homebuilt prism-type total-internal-reflection (PTIR) inverted fluorescence microscope (Olympus IX 71) as described earlier (41–43). Freshly annealed stock partial duplex DNA labeled with Cy3, Cy5 and biotin were diluted to 20–25 pM using the buffer of 10 mM Tris–HCl (pH 7.5) and 100 mM KCl. NeutrAvidin (50 μg/ml) were added to the premade microfluidic chamber and incubated for 2–3 min. After washing, the diluted DNA was added to the chamber and allowed to be immobilization for 1–2 min. Then free DNA was removed by same buffer. All the smFRET measurements were carried out at room temperature (∼23°C ± 2°C) with in an imaging buffer containing 10 mM Tris–HCl (pH 7.5), 100 mM KCl, 1 mM MgCl_2_, 10% glycerol with an oxygen scavenging system (10 mM trolox, 0.5% glucose, 1 mg/ml glucose oxidase and 4 μg/ml catalase) to avoid blinking and to improve dyes stability. All smFRET measurements which produced FRET histograms, single molecule traces, dwell time analysis and heatmap presented in the manuscript were repeated at least three times either in same channel by following the regeneration methods or in a new channel on different days (43).

### smFRET data acquisition and analysis

A solid state of either 532 or 634 nm diode laser (Compass 315M, Coherent) was used to generate an evanescent field through PTIR to excite the fluorophores (Cy3 or Cy5) at the sample chamber. The fluorescence from Cy3 (donor) and Cy5 (acceptor) were simultaneously collected using a water immersion objective and finally projected onto the EMCCD camera (Andor) after passing through the dichroic mirror (cut off = 630 nm). Data were recorded with 100 ms frame integration time, then processed by IDL script (http://www.exelisvis.co.uk/ProductsServices/IDL.aspx) and finally analyzed by MATLAB scripts (https://www.mathworks.com/).

Basic data analysis was carried out by scripts written in MATLAB, and all data fitting were generated by Origin 2018 (https://www.originlab.com/). The FRET efficiency was calculated using *I*_A_/(*I*_D_ + *I*_A_), where *I*_D_ and *I*_A_ represent the intensity of donor and acceptor respectively. Single-molecule FRET histograms were generated from >4000 molecules (21 frames of 20 short movies) collected from different imaging surfaces. Alternating lasers (green and red) were used to excite sequentially both Cy3 and Cy5 (10 frames for Cy3, 1 frame dark and 10 frames for Cy5) to exclude the donor-only molecules from the histogram at the low FRET region. Additionally, the donor leakage was corrected based on the FRET values of donor-only molecules. The smFRET histograms were normalized and fitted by multi-peak Guassian distribution with unconstrained peak position. MATLAB code was used to measure the folding dwell time and then single-exponentially fitted in Origin 2018 to extract decay time.

### Purified protein used in this study

*Escherichia coli* Rep was purified and Rep crosslinking to Rep-X was described previously (30,44). Recombinant human POT1 protein was expressed in a baculovirus/insect cell system and was purified as previously described (35,45). The hexahistidine sumo-tagged TPP1 (amino acids 89–334) was expressed in *E. coli* cells and purified as previously described (35,46). Lambda (λ)-exonuclease protein from *E. coli* was purchased from New England BioLabs (NEB), Cat. No. M0262S, Lot No. 10094819 and certificate of analysis confirm ≥95% protein purity. SDS-PAGE gel image of all used protein shown in Supplementary Figure S1.

### Real-time vectorial folding

The smFRET Rep-X or λ-exonuclease mediated vectorial folding assays were carried out with a micro-fluidic imaging flow chamber (40). A small piece of a plastic reservoir was placed above one hole at one end of the chamber and the other hole at the opposite end was connected to a silicone tube with a syringe pump (Harvard Apparatus, MA). Rep-X (10 nM) with ATP (1 mM or less) suspended in an imaging buffer was loaded into the reservoir. The real-time FRET images were collected by passing solution through the imaging chamber via silicone tubing at a flow rate of 20 μl/s. For C2 and POT1 binding, Rep-X, ATP and C2 or POT1 was added together using the syringe flow system while acquiring a movie. The Rep-X and λ-exonuclease mediated vectorial folding and C2 or POT1 binding. The smFRET time trajectories were analyzed using MATLAB scripts. Using the individual single-molecule real-time flow traces, FRET flow heatmaps were generated. For Rep-X induced vectorial folding, the heatmaps were generated by synchronizing the low to high FRET transition and for λ-exonuclease mediated vectorial folding, the heatmaps were generated by combining of smFRET traces (no synchronization). The FRET heatmaps were generated by an in-house MATLAB script overlaying >100 traces. C2 or POT1 binding kinetics were calculated from the moment of folded state (high FRET) to the moment of first irreversible FRET decline. In each case, >100 molecules were incorporated. For slow C2 or POT1 binding, used the bound fraction calculated from FRET histograms taken over time to calculate the binding kinetics.

## RESULTS

### Vectorial folding of telomere overhang leads to different conformations.

**Figure 1.**
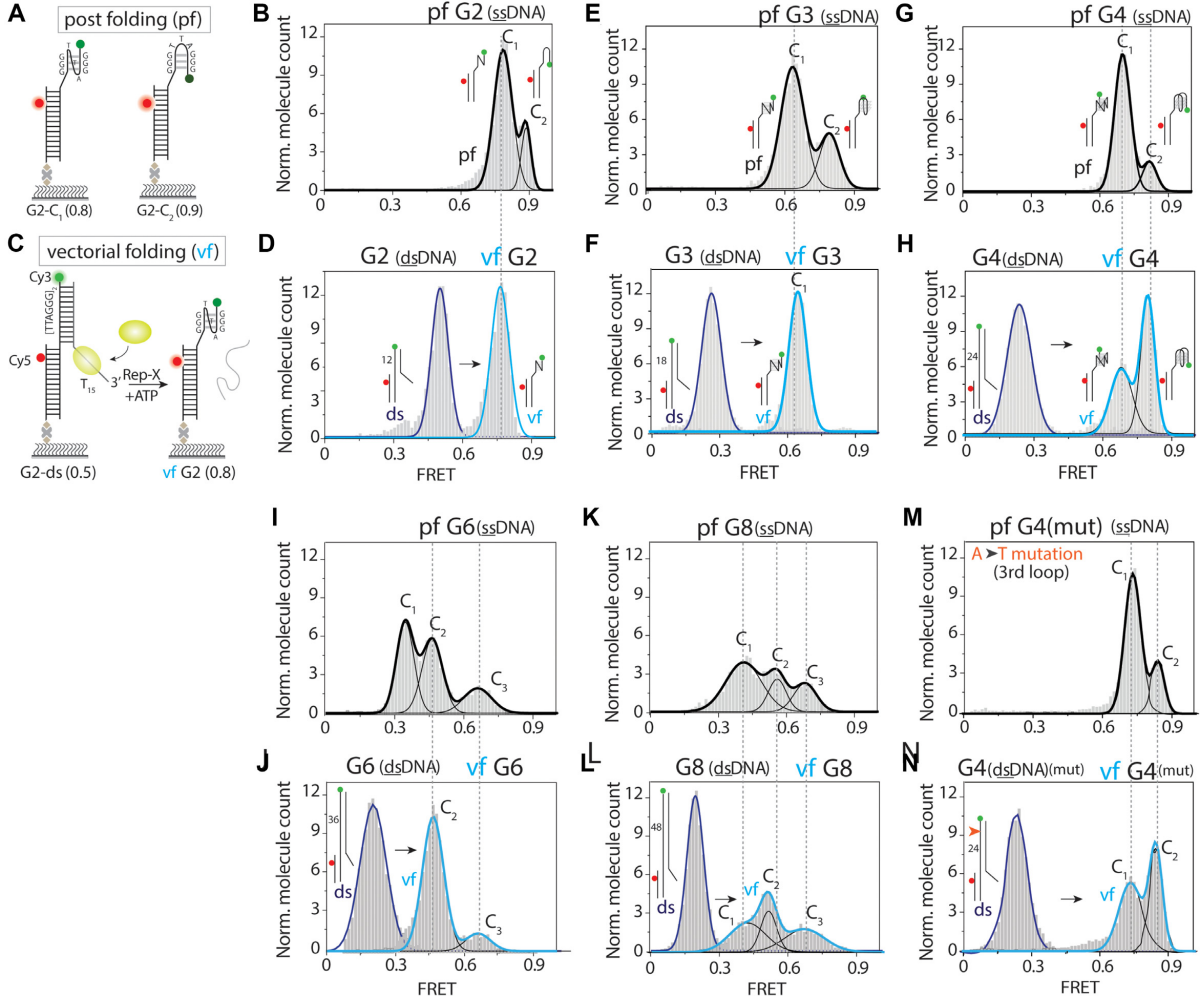
Vectorial folding of telomere overhang leads to different conformations from post folding. (**A**) Schematic diagram of smFRET constructs depicting post folding (pf) of telomere overhang, G2 or (TTAGGG)_2_. (**B**) FRET histogram of pf-G2 showing two FRET peaks (G2-C_1_, ∼0.75 and G2-C_2_, ∼0.9). (**C**) Schematic diagram of smFRET construct depicting vectorial folding (vf) of G2. The unfolding of telomere-duplex via Rep-X (10 nM and 1 mM ATP) leads to subsequent folding of telomere overhang. (**D**) FRET histogram of double stranded G2 (left) showing FRET ∼0.5 and vf-G2 (right) displaying only one FRET peak ∼0.75 (G2-C_1_). (**E–N**) FRET histograms of pf-G3 (**E**), -G4 (**G**), -G6 (**I**), -G8 (**K**), mutated G4 (**M**) and vf of G3 (**F**), G4 (**H**), G6 (**J**), G8 (**L**), mutated G4 (**N**) telomere overhangs. All the vf histograms shown here are taken after 3 min of Rep-X induced unfolding and on each histograms C_1_, C_2_, C_3_ stands for different conformational states. Each FRET construct has a donor (Cy3) dye at 3′ end and acceptor (Cy5) dye positioned in-between the fourth and fifth base pair from the double-/single-strand (ds/ss) junction.

Telomere overhang of tandem TTAGGG repeats readily folds into G4 structures in a buffer that contains NaCl or KCl. Hereafter, we refer to this as post-folding (**pf**). In telomerase extension, however the telomere overhang is folded into G4 structure as it is synthesized in 5′ to 3′ direction by an actively extending telomerase, which we refer to as vectorial folding (**vf**). We employed smFRET to test if the telomere G4 folding resulting from pf and vf differs in conformation and stability. We prepared a series of FRET-labeled partial duplex DNAs (18 bp) with two to eight TTAGGG repeats either in a single strand for pf or in a duplex for vf which is immobilized via biotin-NeutrAvidin to a slide. The donor (Cy3) and acceptor (Cy5) fluorophores were positioned such that the G4 folding induced by the Rep-X unwinding will result in FRET increase (Figure 1). All experiments were carried out in a physiological salt concentration containing 100 mM KCl and 1 mM MgCl_2_ unless stated otherwise. FRET histograms were built by collecting FRET values from >4000 molecules in 20 different fields of view.

In pf, two TTAGGG repeats (G2) of telomere overhang displayed two FRET peaks at ∼0.75 and ∼0.9 indicating two different conformations (Figure 1A, B). The vf was performed by adding Rep-X (10 nM with 1 mM ATP) which shifted a FRET peak at ∼0.5 corresponding to the duplex state to a single FRET peak at ∼0.75 (Figure 1C, D). Similar to G2, the pf of G3 overhang showed two FRET peaks of ∼0.65 and ∼0.8 whereas the vf resulted in only one FRET peak at ∼0.65 (Figure 1E, F). All FRET values obtained for pf and vf of G2 and G3 overhangs are higher than that of the unstructured poly-thymine of the equivalent length, indicating a compact structure (Supplementary Figure S2). Interestingly, the pf and vf of G4 both showed two similar FRET peaks at ∼0.7 and ∼0.85, yet with different relative populations and equilibration time scales (Figure 1G, H). It takes ∼3 h of incubation for the vf to reach the distribution of pf (Supplementary Figure S2). This conformational shifting likely indicates slow relaxation to reach the thermodynamic equilibrium.

To test if the overhang sequence is responsible for generating different folding behaviors between pf and vf, we changed the sequence composition of the G4 overhang first by modifying the loop (TTA), followed by the guanine (GGG). The loop mutation, TTT (instead of TTA) showed the same difference between pf and vf as seen in G4, suggesting that the loop sequence is not a main factor behind the differences between the pf and vf (Figure 1M, N). In contrast, G mutations (G to A, C, T) or chemical modifications of G (8oxoG, O6mG) at either the first or the third G of the 3′ terminal repeat led to vf yielding the same folding as the pf (Supplementary Figure S3). This likely arises because the modified G bases disrupt Hoogsteen base pairing which is important for generating the folding pattern (47). Interestingly, the conformational difference between the pf and vf for G2 to G4 disappeared in NaCl or LiCl-containing solutions, indicating the role of KCl in enabling and dictating the unique vf conformation (Supplementary Figure S2). In longer repeats, G6 and G8 overhang, we still observed different folding pattern between pf and vf although both displayed more complicated conformational states (Figure 1I–L). We note that during the vectorial folding, the number of DNA molecules remained constant since Rep-X does not unwind the DNA substrate on surface due to the folded state of the telomere overhangs (Supplementary Figure S4). Altogether, the vf and pf of telomere overhang lead to different conformations in KCl.

### Real-time smFRET traces reveal folding intermediates.

**Figure 2.**
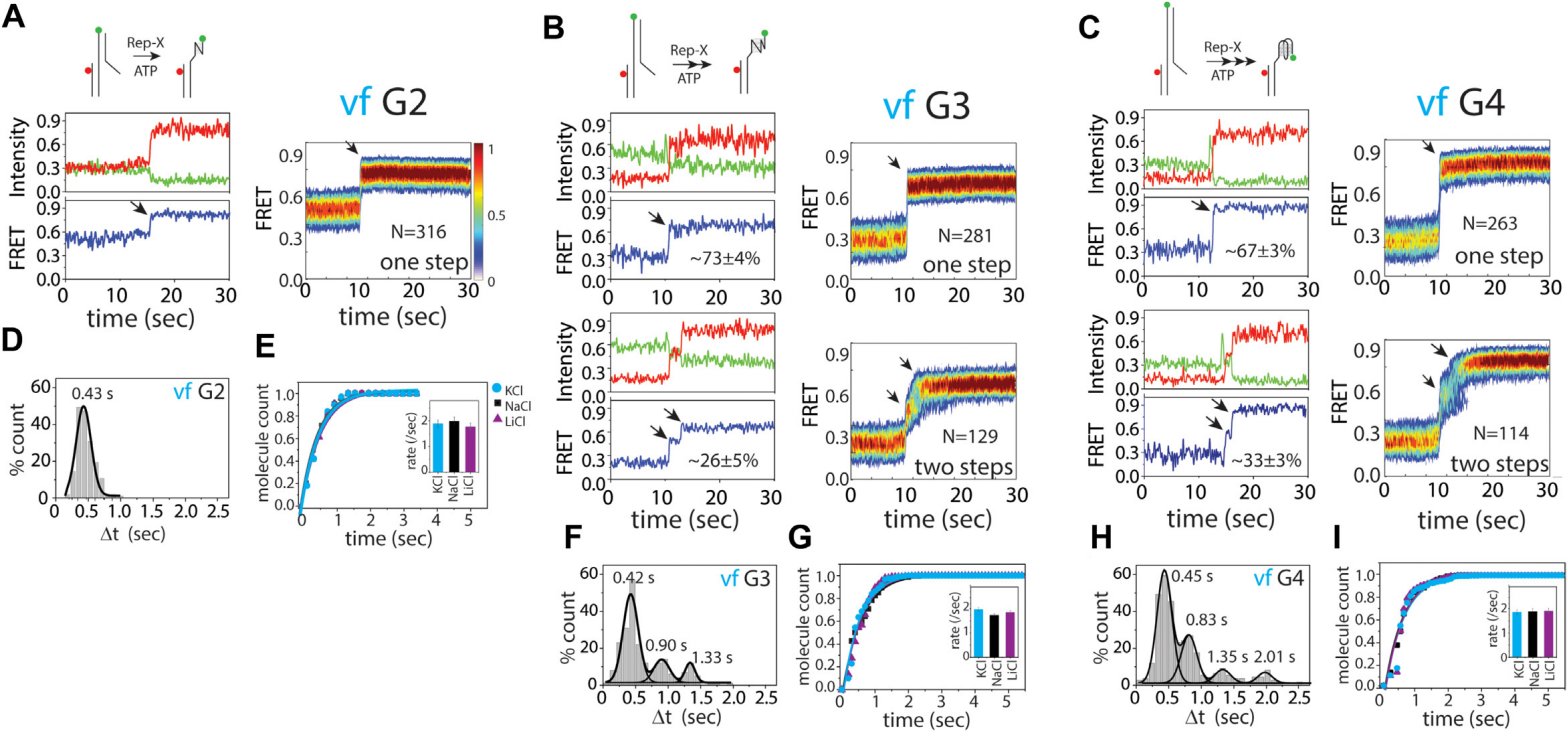
Real-time smFRET traces reveal folding intermediates. (**A–C**) Representative smFRET traces displaying unwinding of telomere duplex (by Rep-X) and subsequent folding of telomere overhangs (green, red and blue colors represents the donor, acceptor and FRET efficiency). Heatmaps were generated by combining smFRET traces that are post-synchronized at the low to high FRET transition during the vf. The number of traces (N) used to generate the heatmaps are included. G2 overhang (**A**) only shows one step folding whereas G3 (**B**) and G4 (**C**) overhang shows multiple steps of folding (indicated by arrows). For G3 and G4, the percentage one and two step traces folding are included. (**D, F, H**) Gaussian fit of the folding dwell time of G2 (**D**), G3 (**F**) and G4 (**H**). (**E, G, I**) The single-exponential fitting of telomere overhang folding for G2 (**E**), G3 (**G**) and G4 (**I**) and the bar graph (inside) indicating the folding rates at three different salts, KCl, NaCl and LiCl.

To investigate the process of vf, we examined the real-time smFRET traces. Upon addition of Rep-X and ATP to G2-dsDNA at ∼10 s, the initial FRET value ∼0.5 FRET from the duplex nearly instantaneously increased to ∼0.75 without displaying any distinguishable intermediate FRET states (Figure 2A). We combined many single-molecule time traces to generate a heatmap in which each trace was synchronized at the moment of FRET increase (Figure 2A). The abrupt transition from duplex to the high FRET state supports a rapid one step folding process.

Unlike in G2, the vf of G3 displayed ∼27% molecules displaying an intermediate FRET state while the remaining (∼73%) displayed one step folding (Figure 2B). The vf of G4 also exhibited ∼67% showing one step and ∼33% displaying two steps of folding (Figure 2C). Since Rep-X unwinds and releases the TTAGGG repeats in 5′ to 3′ direction, our observation of one step folding seen across G2-G4 suggests that the folding starts upon the release of the 3′ end or that the folding starts from the 3′ end, which is consistent with a previous finding (27). It is possible that the population of two step folding may be due to the 5′ initiated folding. The same pattern of steps, single or two step folding, was also observed in vf performed in NaCl and LiCl (100 mM), suggesting that the folding pathway is unaffected by the ionic condition (Supplementary Figure S5).

The shortest folding time was almost identical for G2, G3 and G4 (Figure 2D, F, H). To decipher the meaning of this folding time, we tested the vf of c-Myc sequence which exclusively folds into one configuration. Here, the c-Myc folded in one step with the same dwell time (∼0.41 s) as in G2–G4 (Supplementary Figure S6). Therefore, the shortest FRET increase in G2 to G4 likely correspond to the simplest, likely energetically favorable folding. Interestingly, longer dwell times are observed in G3 and G4, which indicates different durations of intermediate states during the vf process (Figure 2F, H). Again, the same dwell times were obtained in NaCl and LiCl (Supplementary Figure S5). The overall folding pattern for G2, G3 and G4 were also comparable in NaCl and LiCl (Figure 2E, G, I), indicative of vf steps that are independent of ionic condition.

### Vectorial folding rate can be tuned by ATP concentration

**Figure 3.**
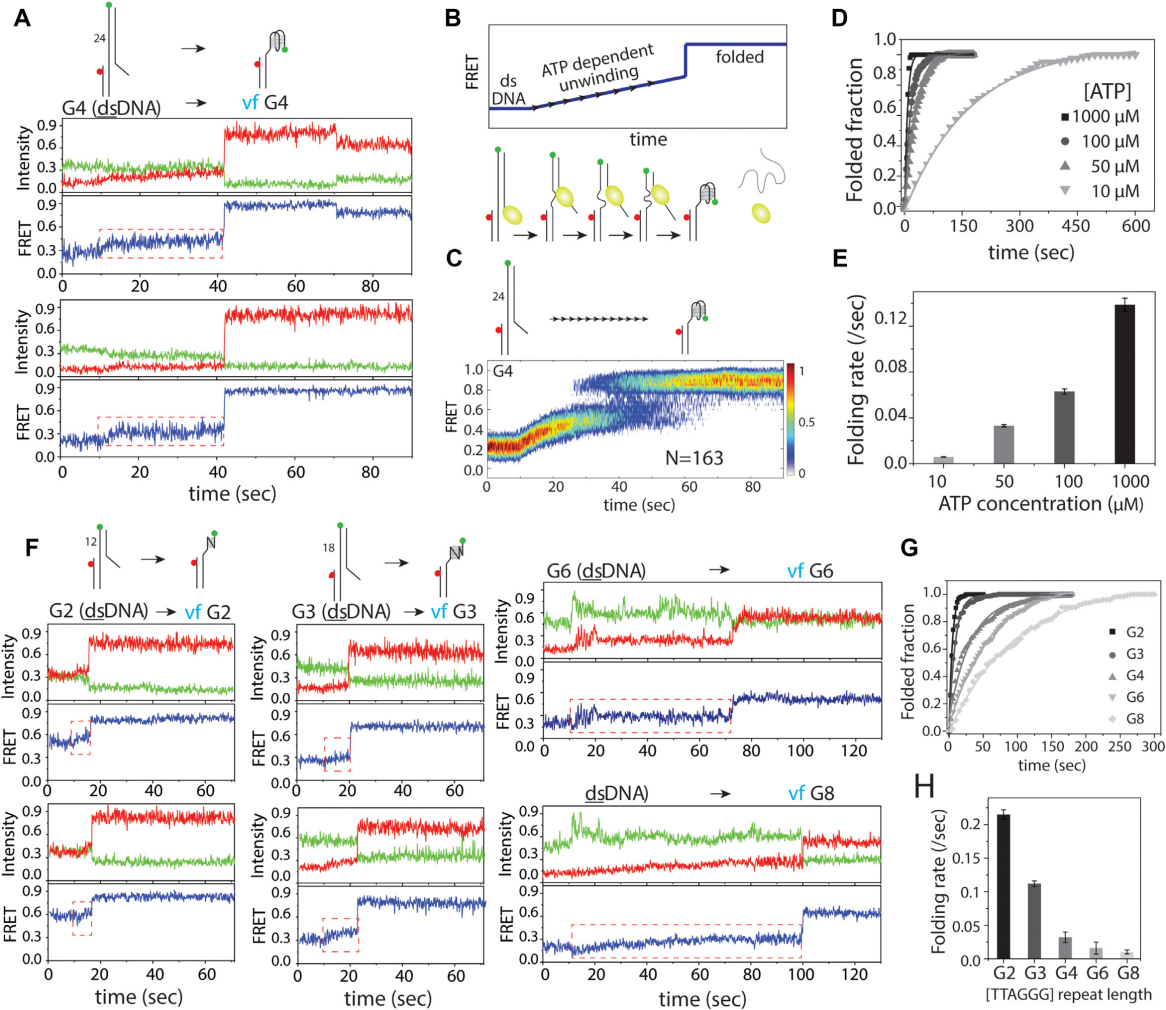
Vectorial folding rate can be tuned by ATP concentration. (**A**) Representative smFRET traces undergoing vf at 50 μM ATP. (**B**) Schematic diagram of Rep-X induced ATP dependent gradual unwinding from dsDNA that leads to a folded telomere overhang. (**C**) Heatmap generated by combining smFRET traces (N = 163) that are synchronized at Rep-X and ATP addition (∼10 sec) which shows a gradual FRET increase followed by G4 folding. (**D**) Single-exponential fitting of G4 folding fraction at different ATP concentrations and the corresponding folding rate (**E**). (**F**) Representative smFRET traces showing vf of G2, G3, G6 and G8 at 50 μM ATP concentration. Red dashed box indicates the gradual FRET increase. (**G**) Single-exponential fitting of G2 to G8 folding fraction at 50 μM ATP concentration and the corresponding rates represented as a bar graph (**H**).

So far, we used a high concentration of ATP (1 mM) which induces fast unwinding by Rep-X, resulting in rapid vectorial folding. Since we use Rep-X to mimic the vectorial release of overhang during telomere synthesis, we lowered the ATP concentration to slow down the Rep-X unwinding to match the speed of the telomerase extension (32,33). At low ATP concentrations, real-time smFRET traces displayed a gradual FRET increase to a certain level (Figure 3A, marked in red dash box) followed by an abrupt increase to a high FRET value. The gradual FRET increase likely corresponds to a gradual release of the overhang strand by a slowly moving Rep-X at low ATP concentrations. Once completely unwound, G4 folds in an abrupt one step, much like in the case of high ATP (Figure 3B). Thus, even with the gradual release of the overhang, the G4 folding was instantaneous, indicating a 3′ end directed folding upon completion of unwinding. Such pattern is prominent in many single molecule traces as indicated by a heatmap (Figure 3C). The folding dwell time was measured by taking the time interval between the low to high FRET transition. We calculated the folding rates at different ATP concentrations by exponentially fitting the dwell time histograms (Figure 3D). As expected, the folding rate was reduced with decreasing ATP concentration (Figure 3E). We found that 50 μM of ATP is the condition at which the folding rate (∼0.039/s) is comparable to that of the telomerase extension ∼1 nt/s/telomerase (48,49), therefore taking about ∼0.04/s for four repeats of 24 nt in length.

We performed vf of G2 to G8 construct at 50 μM of ATP. In all substrates, we observed a similar pattern of a gradual rise in FRET followed by a sudden increase in FRET albeit with some differences depending on the length of repeats (Figure 3F, marked in red dashed line). Despite the different vf rate, the folding conformations were identical to the high ATP concentration (Supplementary Figure S7), suggesting that the folding mechanism is inherent to the vectorial process regardless of the rate of overhang synthesis or release. The folding rate decreased as the number of the TTAGGG repeats increased from G2 to G8 (Figure 3G, H). Interestingly, the folding rates of G4-G8 are on par with the telomerase extension rate stated above (48,49) while the rate is substantially higher for G2 and G3, likely arising from the molecular fraying expected from the short duplex length (Figure 3G, H). Taken together, we show that the vf conducted at the speed of telomerase extension still results in a different conformational state from the pf.

### Vectorial folding leads to an enhanced accessibility

**Figure 4.**
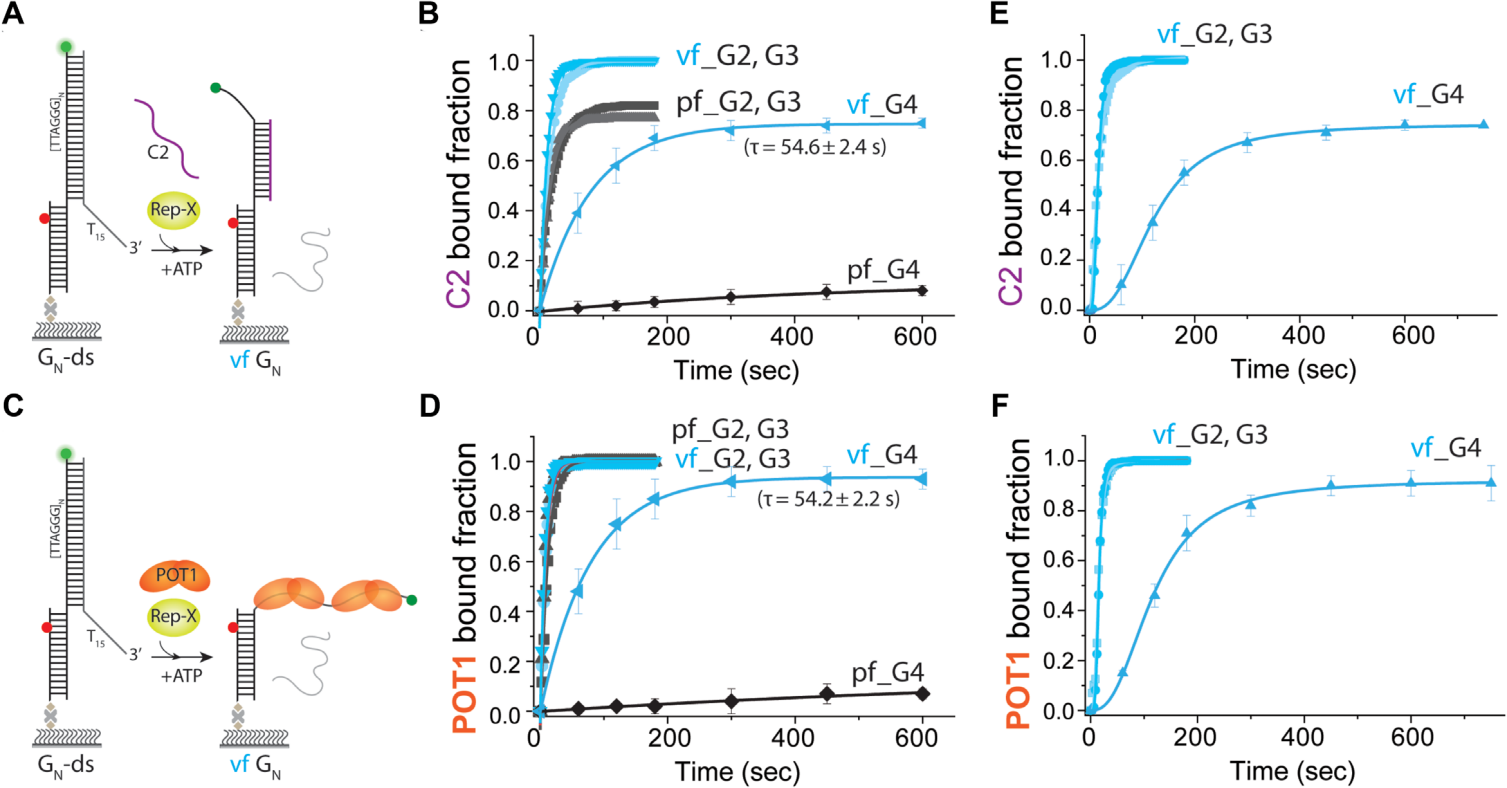
Vectorial folding leads to an enhanced accessibility. (**A**,**C**) Schematic representation of vf and simultaneous annealing of C2 strand, (CCCTAA)_2_ (**A**) or POT1 binding (**C**). (**B, D**) Single-exponential fitting of C2 (250 nM) bound fraction (**B**) or POT1 (500 nM) bound fraction (**D**) by vf and pf at 1 mM ATP concentration. (**E, F**) C2 (250 nM) bound fraction (**E**) or POT1 (500 nM) bound fraction (**F**) by vf at 50 μM ATP concentration.

Next, we asked if the conformations induced by pf vs. vf differ in accessibility to a complementary C-strand ([CCCTAA]2 i.e. C2) and POT1. In our earlier study, we reported that the G4 overhang with chemical modification or mutation becomes more accessible to both C-strand and POT1 than the G4 (47,50). POT1 is a member of the shelterin complex which bind exclusively to telomere overhang with high affinity and sequence specificity (34,35,47). Here, we expect to see a low FRET if C2 anneals to or POT1 binds to the G4 and high FRET if the folded structure is inaccessible to them (Figure 4A, C). For vf, we added Rep-X, ATP with either C2 (250 nM) or POT1 (500 nM) to the vf construct (Figure 4A, C). The resulting FRET histogram for G4 showed ∼70% accessibility for C2 and ∼85% accessibility for POT1 whereas G2 and G3 were 100% became accessible within 3 min (Supplementary Figure S8 and Supplementary Figure S9). Real-time smFRET traces showed a clear transition to high FRET due to the vf, followed by a low FRET resulting from C2 or POT1 binding (Supplementary Figure S8 and Supplementary Figure S9). In contrast, the pf of G4 led to <10% accessibility for both C2 and POT1. G2 and G3 showed ∼80% and 100% accessibility for C2 and POT1, respectively (Supplementary Figure S8 and Supplementary Figure S9). The low accessibility of pf G4 is consistent with our previous results (47,51). The pf-G4 formed in NaCl yields nearly 100% accessibility to POT1 and C2 while pf-G4 formed in KCl renders significantly diminished accessibility (Supplementary Figure S10). Bulk solution study also demonstrated that POT1 affinity for pf-G4 in NaCl was five times higher than for G4 formed in KCl (52). Thus, KCl plays a major role in pf G4 stability. We also note that Rep-X neither alters conformational state nor influences C2 or POT1 binding to both pf or vf G4 (Supplementary Figure S11). For kinetic analysis, we calculated the rate of binding of C2 or POT1 to each DNA constructs based on the smFRET traces (Figure 4B, D). Rates of C2 and POT1 binding to all DNA tested was higher for vf than for pf even for longer lengths (Supplementary Figure S12). In fact, pf-G6 and -G8 form multiple conformations which are dynamically exchanging, hence C2 and POT1 accessibility to pf-G6 and-G8 is significantly higher than pf-G4. Additionally, the POT1 binding accessibility does not change in the presence of the partner protein, TPP1 in both pf and vf G4 (Supplementary Figure S13).

Next, we tested the C2 and POT1 accessibility at 50 μM ATP concentration at which the vf rate matches the telomerase extension speed (Figure 3). Although the initial C2 and POT1 binding was slow due to the low unwinding rate, the maximum bound fraction was comparable to the high ATP condition (Figure 4E, F), again indicating that the accessibility does not depend on the rate of vf. Altogether, we show that the vf leads to a significantly enhanced accessibility to C2 and POT1 binding compared to the pf.

### KCl induced folding does not recapitulate vectorial folding

**Figure 5.**
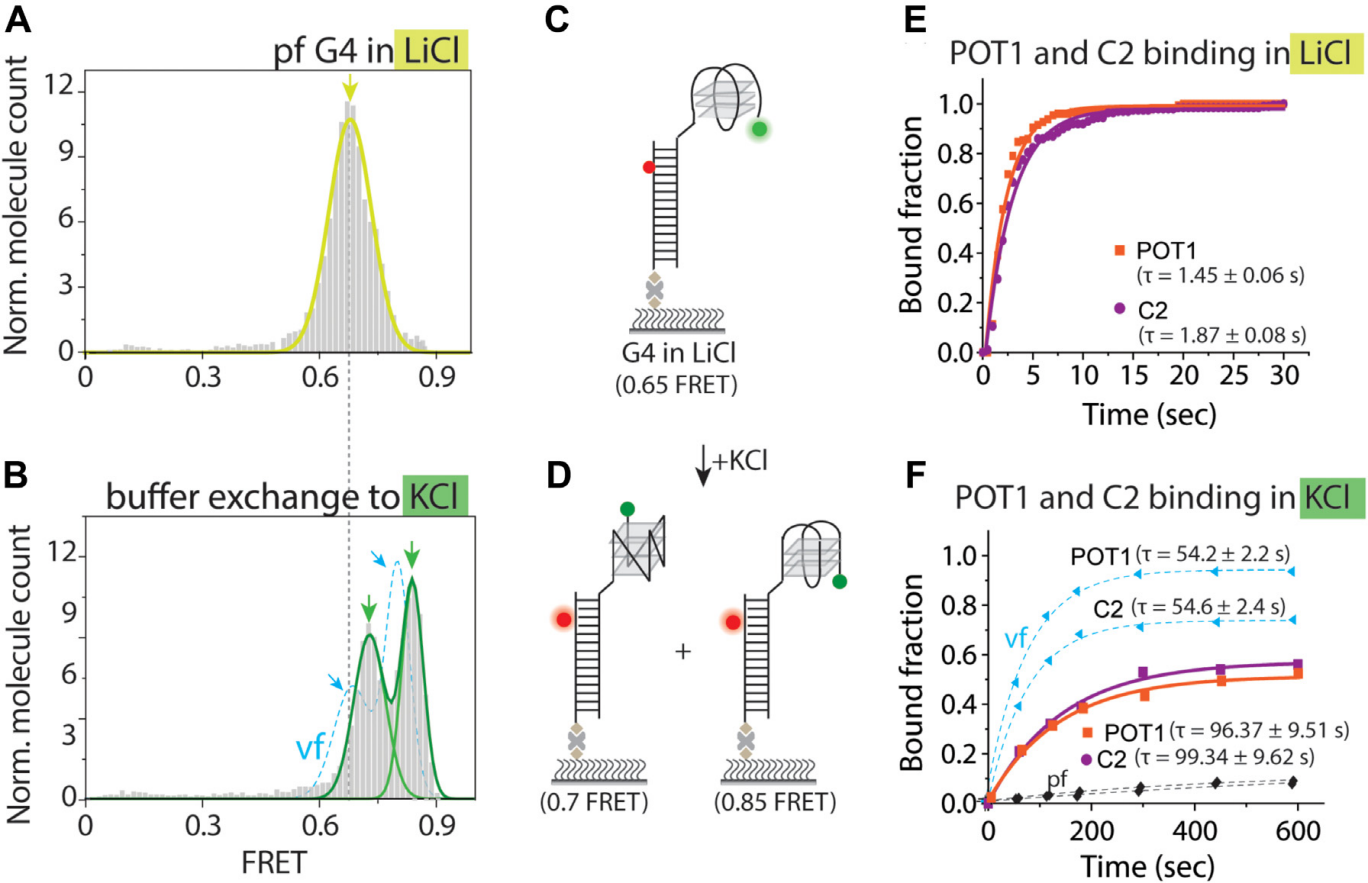
KCl induced folding does not recapitulate the vectorial folding. (**A, B**) FRET histogram of pf-G4 in 100 mM LiCl (**A**) and after the buffer exchange to 100 mM KCl (**B**). The light blue dashed line indicates vf of G4 overhang for comparison. (**C, D**) Schematic depiction of pf-G4 in 100 mM LiCl (**C**) which is in partially folded conditions (FRET ∼0.65). Flow of 100 mM KCl induced G4 folding into two different conformers (FRET ∼0.7 and ∼0.85) (**D**). (**E**) Single-exponential fitting of C2 (250 nM) and POT1 (500 nM) bound fraction on pf-G4 in LiCl. (**F**) Single-exponential fitting of C2 (250 nM) and POT1 (500 nM) bound fraction of pf-G4 in buffer exchange to KCl. The light blue dashed line is the bound fraction during vf by Rep-X.

G4 structure is stabilized in KCl but is much less stable in LiCl buffer (5,16). So far, our results suggest that compared to the pf, the vf leads to different conformations that result in enhanced C2 and POT1 binding. Next, we asked if vf can be recapitulated by a KCl induced folding. We tested this by initially incubating G4 in LiCl which does not support G4 folding, followed by introducing KCl buffer to fold the telomere overhang. In LiCl, G4 showed a mid-FRET peak (∼0.65) which is higher than unstructured poly-T25 (FRET ∼0.35), yet different from pf or vf in KCl (Figure 5A, C and Supplementary Figure S2). Upon replacing the solution with KCl buffer, the histogram shifted to two FRET peaks (∼0.7 and ∼0.85) that are nearly similar to vf, but with different relative populations (Figure 5B, D). To test the KCl induced folding further, we performed the accessibility test by adding C2 (250 nM) or POT1 (500 nM). Despite the similar FRET peaks, the binding rate and maximal bound fraction were both approximately twice lower than in vf (Figure 5E, F and Supplementary Figure S14). Thus, we conclude that KCl induced folding is not equivalent to vf.

### Vectorial folding impact is independent of directionality

**Figure 6.**
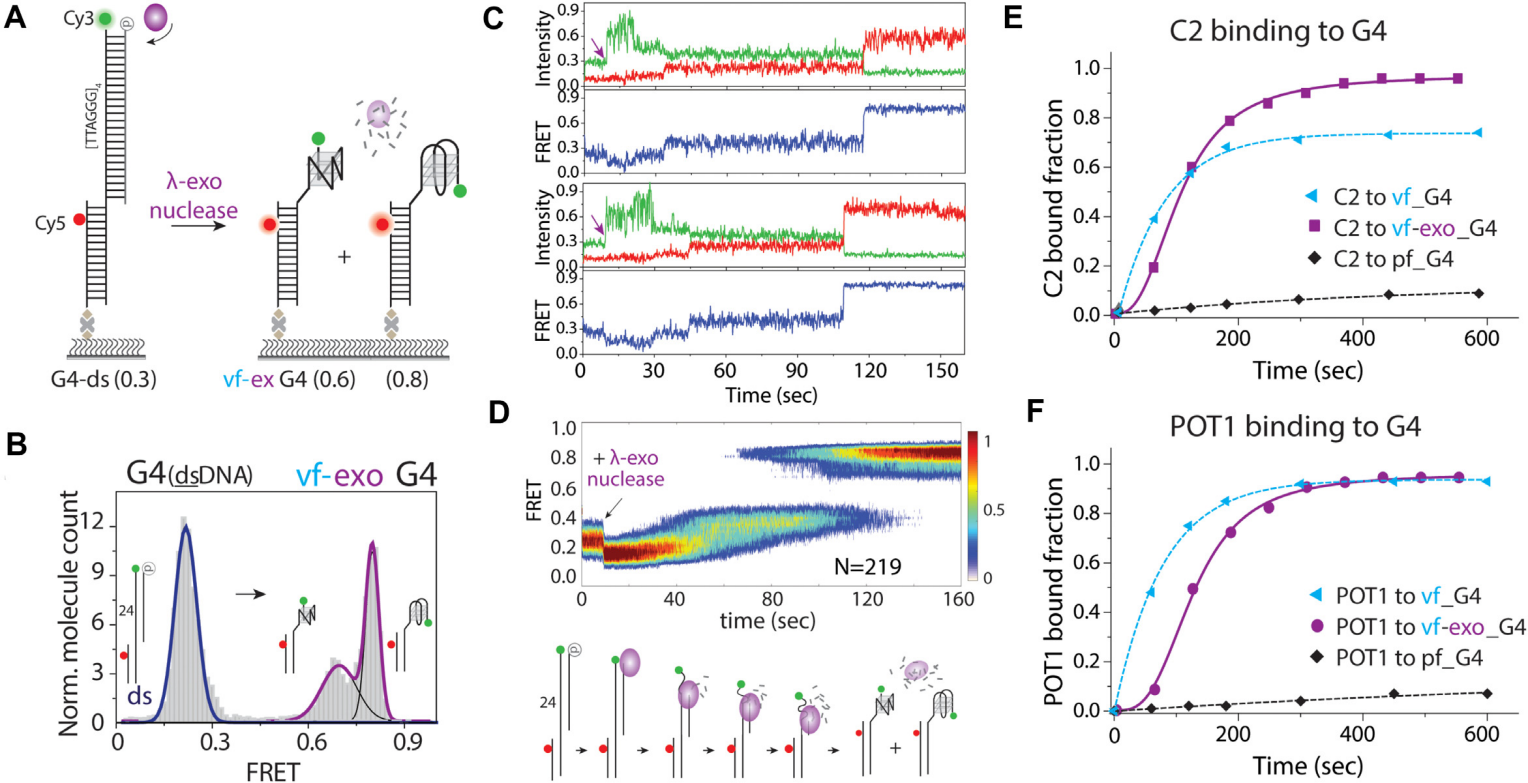
λ-exonuclease mediated vectorial folding leads to different conformation and enhanced accessibility. (**A**) Schematic diagram of smFRET construct used for λ-exonuclease assay. Four repeats of TTAGGG in telomeric duplex has a phosphate group at the C-rich 5′ end. λ-exonuclease digests the C-rich strand in 5′ to 3′ direction, releasing the telomere overhang to fold. (**B**) FRET histogram of before and after λ-exonuclease induced digestion. (**C**) Real-time smFRET traces of λ-exonuclease mediated vf-G4. The arrow indicates the moment λ-exonuclease is applied (∼10 s). (**D**) Heatmap was generated by combining smFRET traces (*N* = 219) without synchronization. The initial λ-exonuclease induced PIFE effect is followed by a gradual FRET increase and G4 folding. A schematic diagram depicting λ-exonuclease induced vf-G4. (**E**, **F**) Single-exponential fitting of C2 (250 nM) and POT1 (500 nM) bound fraction.

So far, we have used Rep-X to follow the directionality of the telomere extension in 5′ to 3′ direction. Next, we asked if the vf effect depends on the directionality of the strand release. To address this question, we took advantage of the lambda (λ)-exonuclease which excises the C strand in the 5′ to 3′ direction such that the telomere overhang opens up in a reverse direction from the 3′ to 5′ end. This process may mimic post-replication process in which blunt ended DNA is nuclease digested to generate the 3′ overhang for genome integrity (1,53) which is under the control of TRF2-bound Apollo nuclease (54,55). For this experiment, we modified our smFRET construct by inserting a 5′-phosphate for λ-exonuclease binding and initiation while keeping the FRET probes at the same positions (Figure 6A). λ-exonuclease digestion is expected to result in vf in an opposite direction (Figure 6A). Like the Rep-X mediated vf, the histogram showed FRET peak at ∼0.2 for the G4–C4 duplex. After the λ-exonuclease treatment, the FRET peak shifted to two peaks that are highly similar to the Rep-X induced vf of G4 (Figure 6B).

To check the vectorial folding mechanism, we looked through the real-time smFRET traces. Upon addition of λ-exonuclease at ∼10 s, there is a Cy3 intensity fluctuation likely due to the PIFE (protein induced fluorescence enhancement) (56,57) effect expected from the initial contact by the λ-exonuclease (Figure 6C). The initial PIFE dependent signal fluctuation is followed by a first phase of FRET increase due to the nuclease digestion and the second phase of FRET increase that indicates G4 folding (Figure 6C). Majority of the traces (∼>95%) showed one step folding from mid FRET to high FRET with the dwell time of ∼0.41 s which is consistent with the Rep-X mediated one-step vectorial folding (Supplementary Figure S15). The heatmap generated by combining smFRET traces displays PIFE signal (FRET appears lower, but this is only due to the PIFE which increases Cy3 without affecting Cy5) followed by a gradual FRET increase and a rapid FRET increase (Figure 6D), which resembles Rep-X mediated vectorial folding at low ATP (50 μM) condition. We observed a similar pattern for G2 and G6 (Supplementary Figure S16). Next, we tested the C2 and POT1 binding accessibility during the exonuclease mediated vf. Both C2 and POT1 bound fractions reached ∼90–95% which is comparable to the Rep-X mediated vf (Figure 6E, F). Real-time smFRET traces showed initial digestion followed by C2 or POT1 binding (Supplementary Figure S15). Overall, the binding kinetic is slighted slower in exonuclease vf, likely due to the differences between the two enzyme systems. The maximum binding of C2 is slightly higher in exonuclease-vf, which may arise from an easier access to the tail end, which is released first by the exonuclease, but last by Rep-X. Altogether, exonuclease mediated vf results in a similar conformations and accessibility to the Rep-X induced vf, strengthening the unique conformation achieved by the vectorial folding process.

## DISCUSSION

The G4 folding of telomere overhang was demonstrated both *in vitro* and *in vivo* (16,58). Four repeats of TTAGGG can potentially fold into heterogeneous conformations including parallel, antiparallel and hybrid (9,11,12). Recently, an *in vitro* study showed that G4 forms on a nascent telomeric DNA during telomerase extension, yet with unknown conformational state (16). The G4 folding process in the context of telomerase extension is inherently different from the usual experimental setting of post folding in two important aspects. First, the G-rich strand is synthesized and released directionally or vectorially. Second, the strand is released and exposed gradually, not all at once. Our result reveals a significant difference between the vf and pf in both conformational state and accessibility in a manner that depends on the presence of potassium and the successive runs of guanine.

The G2 and G3 overhang exhibited higher FRET states than the same length of unstructured poly-thymine (Supplementary Figure S2), indicating a compact structure like a G-hairpin or G-triplex, consistent with a previous finding (29,59). Interestingly, both G2 and G3 folded into two states by pf, likely representing the guanine strand running in the same (parallel) or the opposite orientations. In contrast, vectorial folding led to only one FRET state which matches the lower FRET value of the two states seen in pf (Figure 1). Based on the donor and acceptor separation expected for the two conformations, the FRET value seen in vf corresponds to the parallel arrangement in which the guanine strands are oriented in the same direction. For G4, the two FRET peaks seen in pf is consistent with our previous work (7,47). Interestingly, both Rep-X and exonuclease mediated G4 vf led to primarily non-parallel state which shifted to parallel conformation over hours (Supplementary Figure S2). This pattern clearly indicates that vf kinetically traps G4 in a metastable non-parallel conformation which can slowly rely into a thermodynamically favorable parallel structure (59,60). We consider that the vf, rather than the pf likely depicts the cellular scenario in which telomerase actively synthesizes the telomeric overhang, which in turn, folds into G4 structure.

We found one folding step for G2 and additional intermediate folding steps for the vf of G3 and G4, respectively (Figure 2). Nevertheless, the predominant step observed in all constructs was the single step with a dwell time of ∼0.4 s, which also matched the folding time of the exclusively parallel G4, cMyc. This single step transition likely indicates that the folding initiates from the 3′ end after the completion of the duplex unwinding by Rep-X, which is consistent with a previous finding (6,27). The difference of a slightly longer dwell time (∼0.73 s) reported in a previous study may be due to a longer length of the construct used (27). What gives rise to the intermediate steps observed in G3 and G4? First, Rep-X may take pauses during unwinding, yet this is unlikely based on the high speed of translocation (30). Second, overhang folding may start from the 5′ end. This is a possibility which is also suggested in previous studies (6,27). Third, the stepwise folding may arise from the 3′ end. Fourth, folding intermediates such as G-hairpin or G-triplex may form before the final structure emerges (29). Exonuclease mediated 3′ to 5′ vectorial folding showed only one step folding with an identical one-step dwell time (∼0.41 s), signifying that the one-step folding arises from the vectorial folding process and is independent of directionality. This result also implies that 3′ initiated folding only produces one-step, thus the multi-step folding may arise from the 5′ initiated folding pathway. The detailed mechanism underlying multiple folding steps warrants further study.

We used two orthogonal approaches to mimic the vectorial folding of telomere overhang by employing a superhelicase, Rep-X and the λ-exonuclease. In both cases, the overhang sequence was released gradually one nucleotide at a time. The single nucleotide releases entailed ATP driven unwinding by Rep-X and digestion of 5′ phosphorylated strand by exonuclease. Both processes led to a gradual FRET increase, representing a conversion from a duplex to a single-strand DNA followed by an abrupt FRET spike. We interpreted the second phase of sudden FRET increase as the rapid folding of the overhang sequence. Does the folding seen in G2 and G3 represent intermediate states within the G4 folding? It is unlikely based on our observation that even under low ATP concentration i.e when the release was slowed down to allow more time for intermediate structures to emerge, we still observed an abrupt FRET increase that correspond to rapid one-step folding only upon the full release of all TTAGGG repeats (Figure 3). It is plausible that there is residual interaction between the enzyme and unwound DNA and the C-strand has to fully clear before any folding occurs. This observation is in agreement with a previous study in which G4 formation was observed within an actively extending telomerase complex *in vitro* (16).

**Figure 7.**
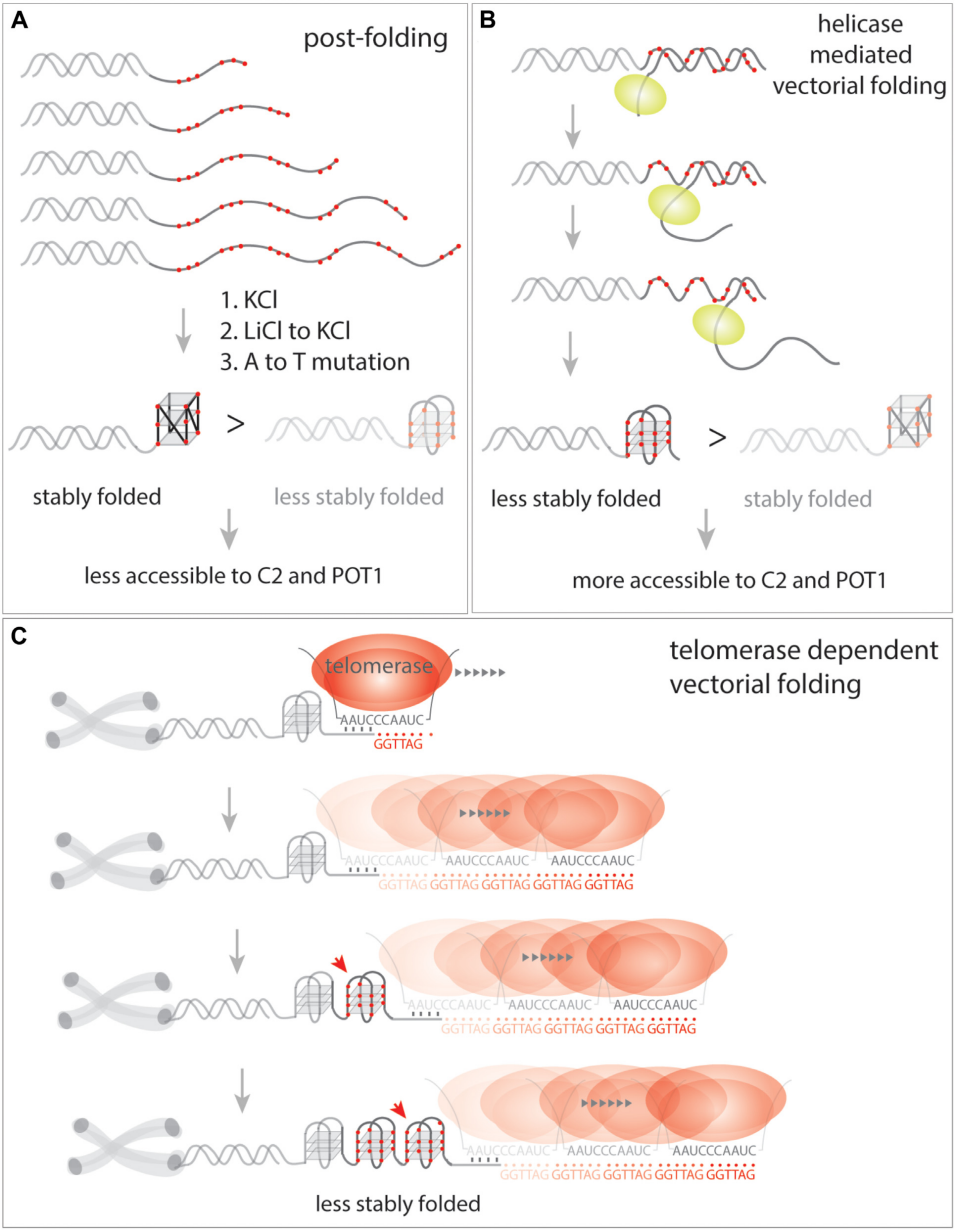
Proposed model. (**A**) Post folding of telomere overhangs results in a stably folded conformation which is less accessible to C2 and POT1 binding. (**B**) Helicase (e.g. Rep-X) mediated vectorial folding of telomere overhangs lead to a less stable folding which is more accessible to C2 and POT1 binding. (**C**) It is expected that telomerase dependent extension of telomere overhang undergoes vectorial folding which may lead to high accessibility for shelterin proteins.

The most biological relevant implication revealed by our study is that the vectorially folded overhang is significantly more accessible to molecules that bind the overhang sequence (Figure 7). While pf and vf of G2 and G3 rendered equal accessibility to both C-rich strand and POT1, G4-vf enabled substantially faster and higher level of binding to C-rich strand and POT1 than G4-pf (Figure 4). In our previous study in which we performed single-molecule assay for detecting the telomerase extension in real time *in vitro*, we used the same C-rich strand ([CCCTAA]2 i.e. C2), to probe the extended overhang (32). In retrospect, the assay was feasible due to the vectorially produced overhang which allowed for binding of C-rich strand. We also tested if such highly accessible structure can emerge from KCl induced folding which is expected to stabilize the G4 structure. Interestingly, when LiCl was replaced by KCl buffer, G4 folding changed to nearly similar conformational states as the case of vf (Figure 5). Nevertheless, the accessibility to C2 and POT1 was markedly compromised, signifying that the reorganization of G4 induced by KCl is not equivalent to the vectorial folding.

Telomeres maintain genomic integrity in normal cells and telomere attrition during successive cell division can induce chromosomal instability (61–63). In majority of cancer cells, telomere length is maintained by upregulating the telomerase activity (18–20). On the other hand, a nuclease activity is required to generate a 3′ overhang after a round of DNA replication (64). In both cases, the overhang is expected to fold vectorially since both processes involve generation of single strand overhang enzymatically (Figure 7). In this regard, our Rep-X mediated vf mimics the telomerase-based overhang synthesis whereas the λ exonuclease induced vf resembles the 3′ overhang generation, both of which are critical for proper genome maintenance. Taken together, we demonstrate that the vectorially extended overhang exhibit an unusually destabilized state that enhances accessibility, which may be critical for reestablishing shelterin bound telomere structure post telomerase extension.

## Supplementary Material

gkac401_Supplemental_File
