# Supplementary material for: Vectorial folding of telomere overhang promotes higher accessibility
Source: Nucleic Acids Res. 2022 Jun 10;50(11):6271–83. doi: 10.1093/nar/gkac401 (PMC9226509; doi:10.1093/nar/gkac401)
Supplement: gkac401_Supplemental_File [file gkac401_supplemental_file.pdf]

SUPPLEMENTARY INFORMATION

Vectorial folding of telomere overhang promotes higher accessibility

Tapas Paul[1], Patricia L. Opresko[2], Taekjip Ha[1,3,4] and Sua Myong[1,3,*]

Author Affiliations:
[1]Department of Biophysics, Johns Hopkins University, Baltimore, MD 21218, USA.
[2]Department of Environmental and Occupational Health, University of Pittsburgh Graduate School of Public Health, and UPMC Hillman Cancer Center, Pittsburgh 15213, USA.
[3]Physics Frontier Center (Center for Physics of Living Cells), University of Illinois, 1110 W. Green St., Urbana, IL, 61801, USA.
[4]Howard Hughes Medical Institute, Johns Hopkins University, Baltimore, Maryland
*To whom correspondence should be addressed: Email: smyong@jhu.edu; Tel: 410-516-5122; Fax: 410-516-4118.

Supplementary information includes:
Supplementary Table 1
Supplementary Figures, S1-S16

Supplementary Table 1. DNA Oligonucleotides (5' to 3') used in this experiment.

G2-Cy3	TGG CGA CGG CAG CGA GGC (TTA GGG)2 /Cy3/
G3-Cy3	TGG CGA CGG CAG CGA GGC (TTA GGG)3 /Cy3/
G4-Cy3	TGG CGA CGG CAG CGA GGC (TTA GGG)4 /Cy3/
G3-TTT GGG-Cy3	TGG CGA CGG CAG CGA GGC (TTA GGG)3 TTT GGG /Cy3/
G3-TTA AGG-Cy3	TGG CGA CGG CAG CGA GGC (TTA GGG)3 TTA AGG /Cy3/
G3-TTA TGG-Cy3	TGG CGA CGG CAG CGA GGC (TTA GGG)3 TTA TGG /Cy3/
G3-TTA 8oxoGGG-Cy3	TGG CGA CGG CAG CGA GGC (TTA GGG)3 TTA 8oxoGGG /Cy3/
G3-TTA O6mGGG-Cy3	TGG CGA CGG CAG CGA GGC (TTA GGG)3 TTA O6mGGG /Cy3/
G3-TTA GGA-Cy3	TGG CGA CGG CAG CGA GGC (TTA GGG)3 TTA GGA /Cy3/
G3-TTA GGT-Cy3	TGG CGA CGG CAG CGA GGC (TTA GGG)3 TTA GGT /Cy3/
G3-TTA GG8oxoG-Cy3	TGG CGA CGG CAG CGA GGC (TTA GGG)3 TTA GG8oxoG /Cy3/
G3-TTA GGO6mG-Cy3	TGG CGA CGG CAG CGA GGC (TTA GGG)3 TTA GGO6mG /Cy3/
G1-TTA TGG-G2-Cy3	TGG CGA CGG CAG CGA GGC TTA GGG TTA TGG (TTA GGG)2 /Cy3/
G1-TTA GGT-G2-Cy3	TGG CGA CGG CAG CGA GGC TTA GGG TTA GGT (TTA GGG)2 /Cy3/
G6-Cy3	TGG CGA CGG CAG CGA GGC (TTA GGG)6 /Cy3/
G8-Cy3	TGG CGA CGG CAG CGA GGC (TTA GGG)8 /Cy3/
C2	(CCC TAA)2
C2-T15	(CCC TAA)2 (T)15
C2-Phos	Phos-(CCC TAA)2
C3-T15	(CCC TAA)3 (T)15
C4-T15	(CCC TAA)4 (T)15
C4-Phos	Phos-(CCC TAA)4
C6-T15	(CCC TAA)6 (T)15
C6-Phos	Phos-(CCC TAA)6
C8-T15	(CCC TAA)8 (T)15
T12-Cy3	TGG CGA CGG CAG CGA GGC (T)12 /Cy3/
T18-Cy3	TGG CGA CGG CAG CGA GGC (T)18 /Cy3/
T25-Cy3	TGG CGA CGG CAG CGA GGC (T)25 /Cy3/
cMyc	TGG CGA CGG CAG CGA GGC TT GGG T GGG TA GGG T GGG /Cy3/
cMyc-com-T15	CCC A CCC TA CCC A CCC (T)15
Cy5-18mer-Bio	GCC T/Cy5/ CG CTG CCG TCG CCA /Bio/

Supplementary Figure 1

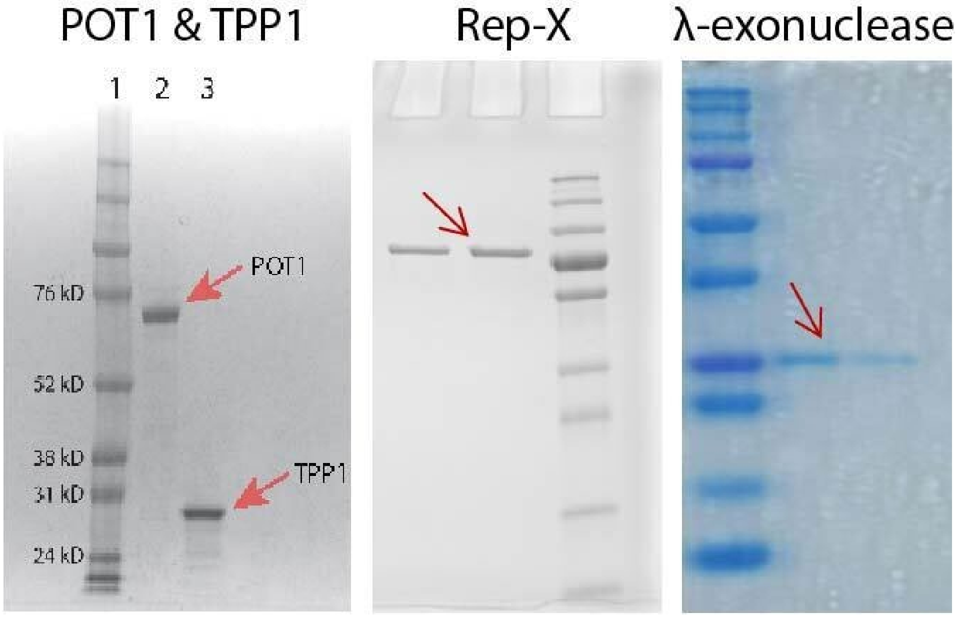

Figure S1: SDS-PAGE gel with coomassie staining of POT1 & TPP1 (left side), Rep-X (middle) and lambda (λ)-exonuclease (right side). Lambda (λ)-exonuclease protein has >95% purity from New England BioLabs (NEB), Cat. No. M0262S, lot number: 10094819.

Supplementary Figure 2

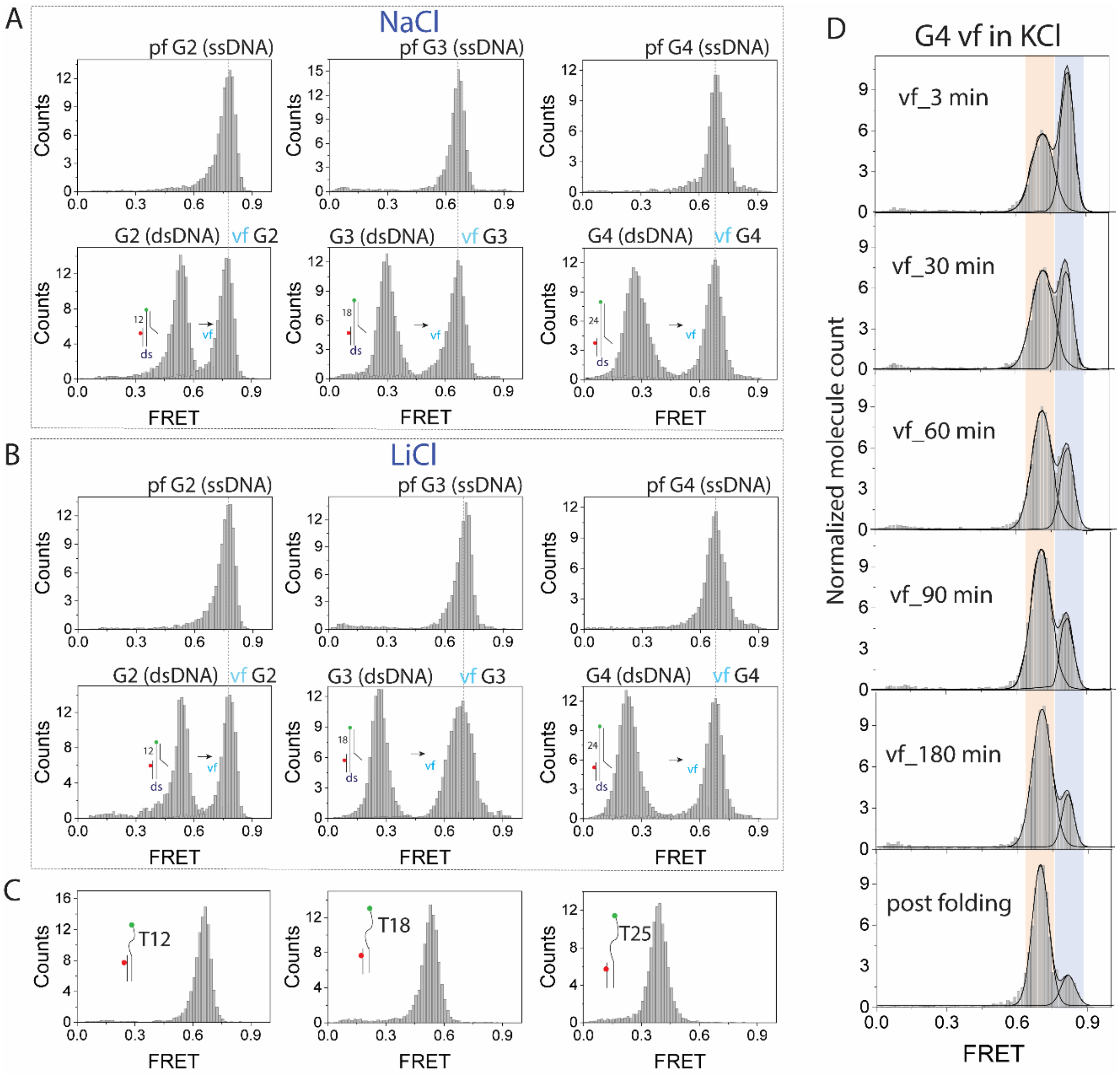

Figure S2: **(A)** The FRET histograms of post folding (top) and vectorial folding (bottom) of G2, G3 and G4 telomere overhangs in 100 mM NaCl salt containing buffer. **(B)** The same FRET histograms like (A) of post folding and vectorial folding in 100 mM LiCl. In both cases, the vectorial folding was performed by unfolding of telomere-duplex using 10 nM Rep-X and 1 mM ATP. All the vectorial folding histograms shown here at 3 minutes of folding. **(C)** The FRET histogram of non-structure poly T12, T18 and T25 in 100 mM KCl salt containing buffer. The same FRET peak was also observed at 100 mM NaCl and LiCl. Those poly-T is comparable with the same number of nucleotides of G2, G3 and G4 telomere overhang. **(D)** The FRET histogram of G4 telomere overhang during vectorial folding with time. High FRET peak population decreases and simultaneously mid FRET peak population increases with time and after 180 minutes the FRET histogram look almost similar to post folding condition (bottom).

Supplementary Figure 3

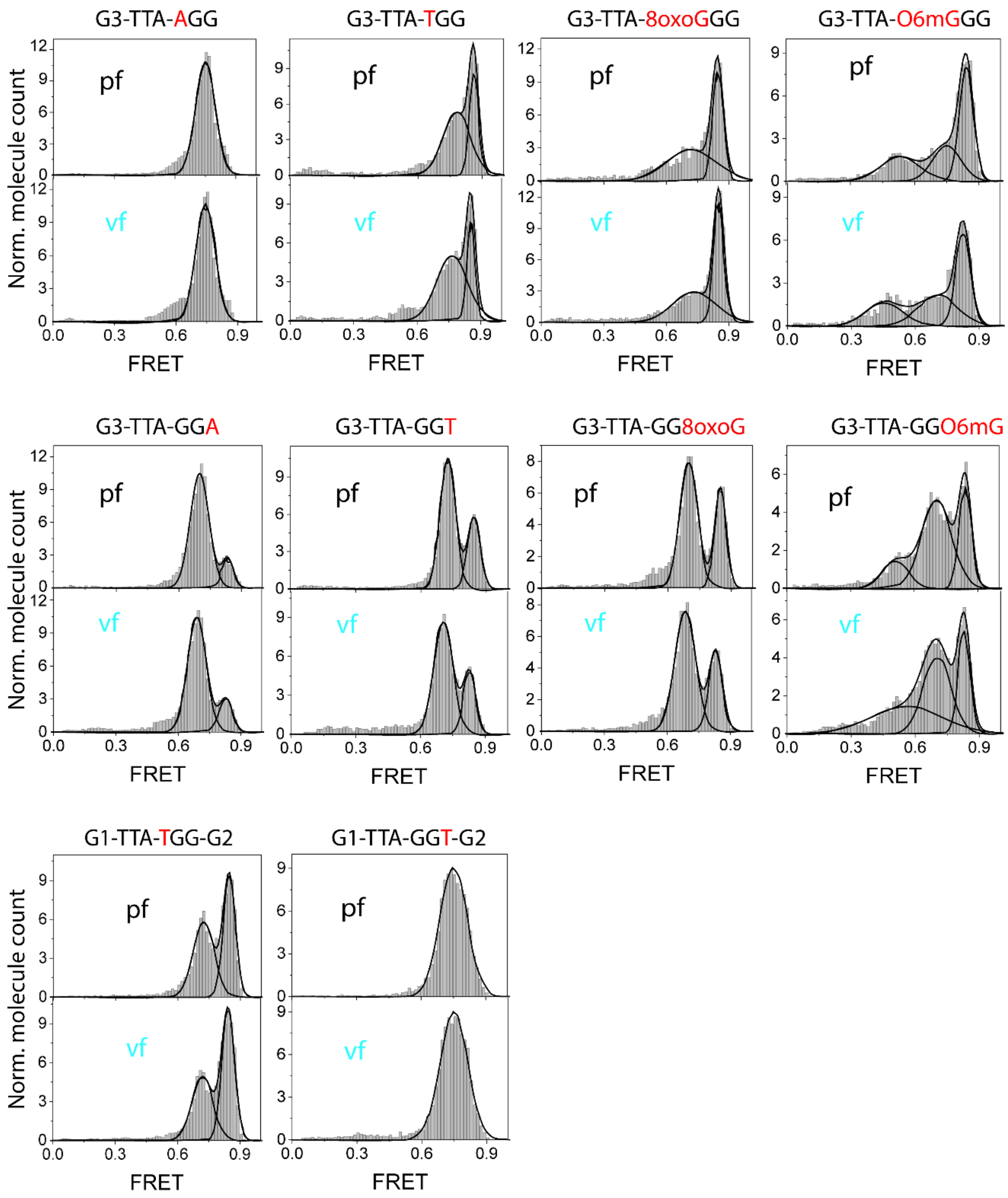

Figure S3: The FRET histograms of post folding and vectorial folding of mutated and damaged (mentioned in red color) G4 telomere overhang in KCl. The top and middle histograms are the 1st and 3rd guanine mutation or damage in the fourth TTAGGG repeat of the G4 overhang, respectively. The bottom histograms are the 1st and 3rd guanine mutation (G to T) in the second TTAGGG repeat of the G4 overhang, respectively.

Supplementary Figure 4

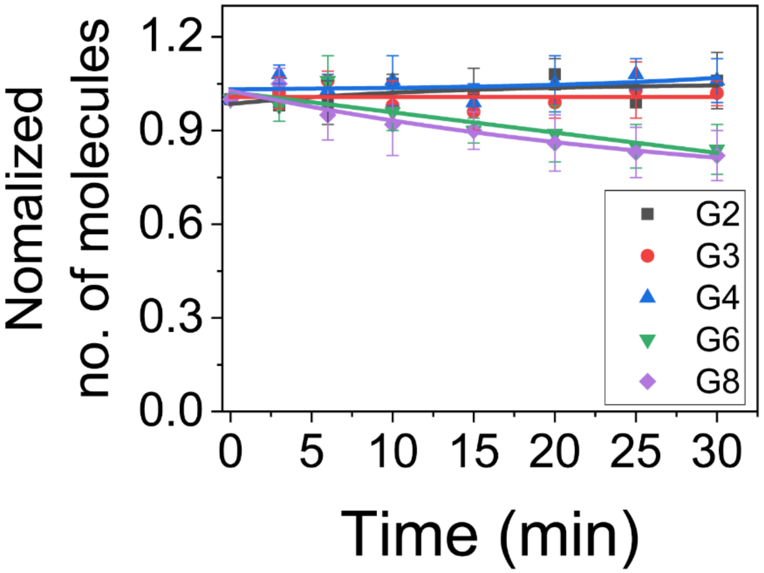

Figure S4: Rep-X translocating through C-rich strand and releasing the G-rich strand which facilitate to fold vectorially. Since, Rep-X present in solution, may bind to released G-rich strand and unwind the immobilized partial duplex which leads to disappearance of Cy3 signals and concomitant loss of FRET. Hence, molecule counted during vectorial folding (using 10 nM Rep-X and 1 mM ATP in 100 mM KCl) of G2 to G8 overhangs. The counted number of molecules remain almost constant with time indicating Rep-X unable to bind and unwind the telomere overhangs which further confirm the folding nature of telomere overhangs.

Supplementary Figure 5

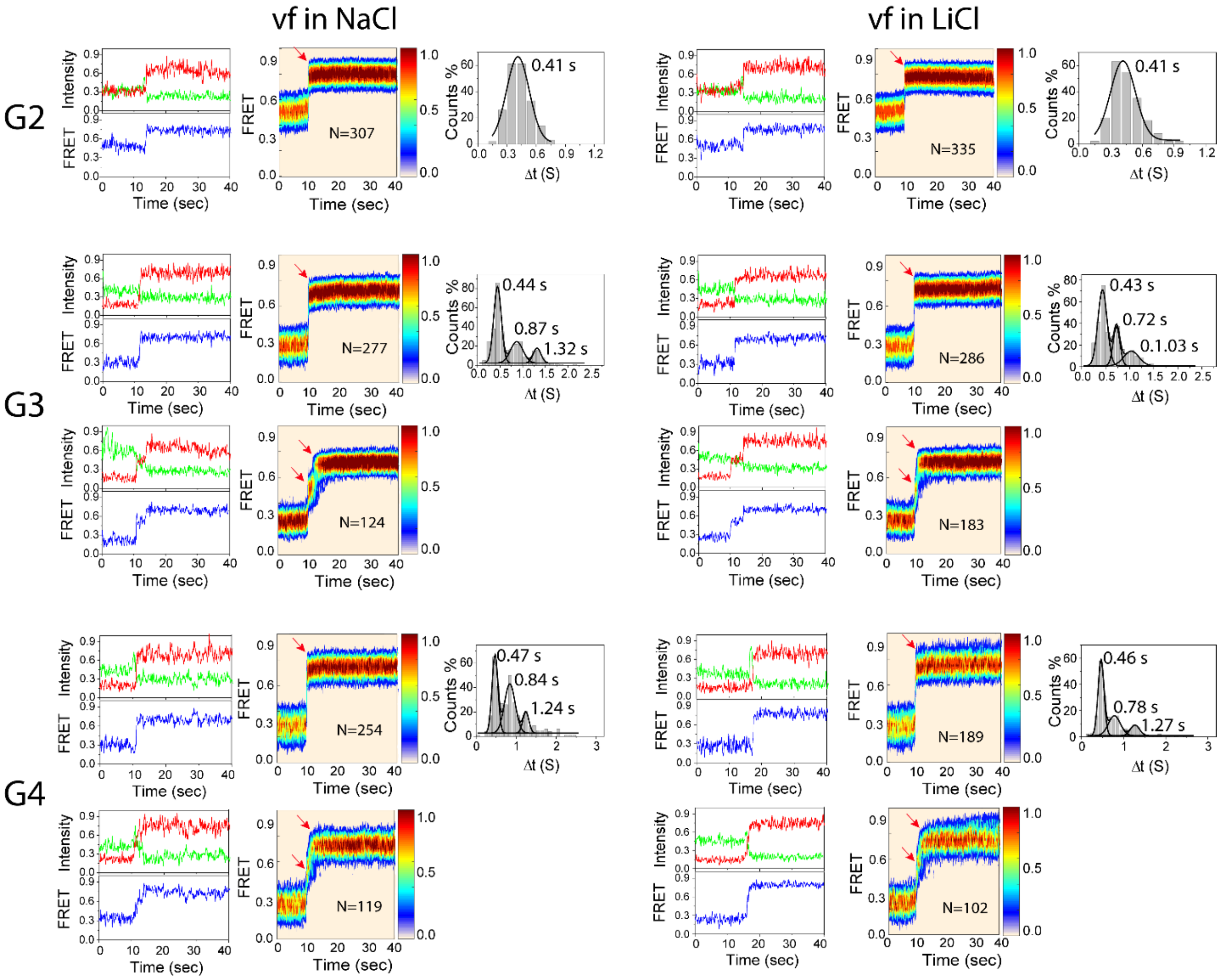

Figure S5: Representative real-time smFRET traces showing unwinding of telomeric duplex and folding of G2, G3 and G4 telomere overhangs respectively in 100 mM NaCl or 100 mM LiCl. Besides, the heatmap generated by combining the smFRET traces, synchronizing at the low to high FRET transition during vectorial folding. The number of traces (N) used to generate the heatmap are mentioned accordingly. As in vectorial folding at 100 mM KCl, G2 overhang show one step folding where as G3 and G4 overhang show one and two steps of folding (indicated in arrow) in both salt condition. Gaussian fit of the folding dwell time of G2, G3, and G4 telomere overhangs in both salt condition.

Supplementary Figure 6

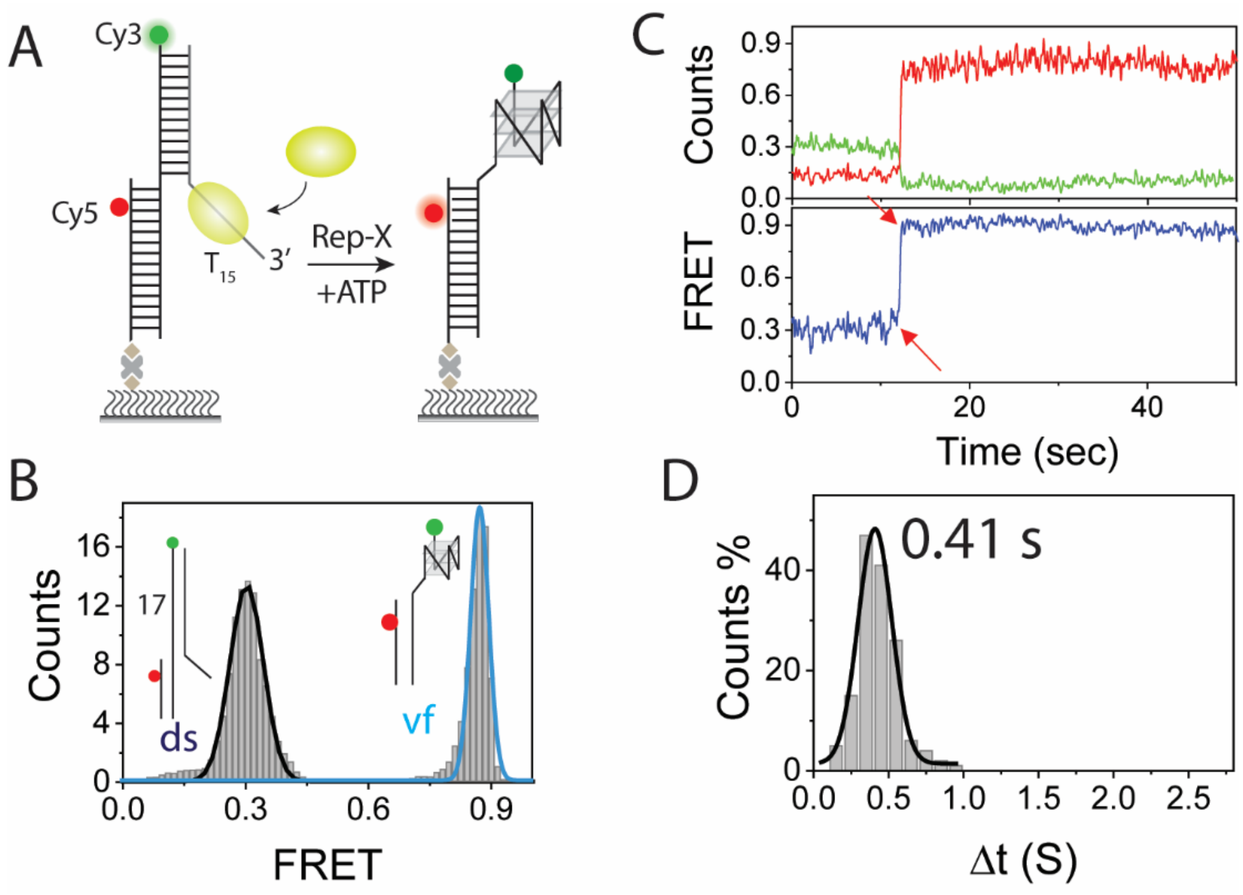

Figure S6: (A) Schematic smFRET diagram of vectorial folding where unfolding of c-Myc-duplex via Rep-X (10 nM and 1 mM ATP) and subsequent folding of parallel c-Myc G-quadruplex. **(B)** The FRET histogram of c-Myc-duplex (FRET ~0.3) and vectorial folding of c-Myc GQ (FRET ~0.85). **(C)** The representative real-time smFRET trace showing unwinding of c-Myc-duplex (by Rep-X) and folding of c-Myc GQ. The red arrow indicates the unfolding to one step folding. **(D)** Gaussian fit of the folding dwell time (~0.41 s) which is comparable to the one step vectorial folding of telomere overhang (G2, G3 and G4).

Supplementary Figure 7

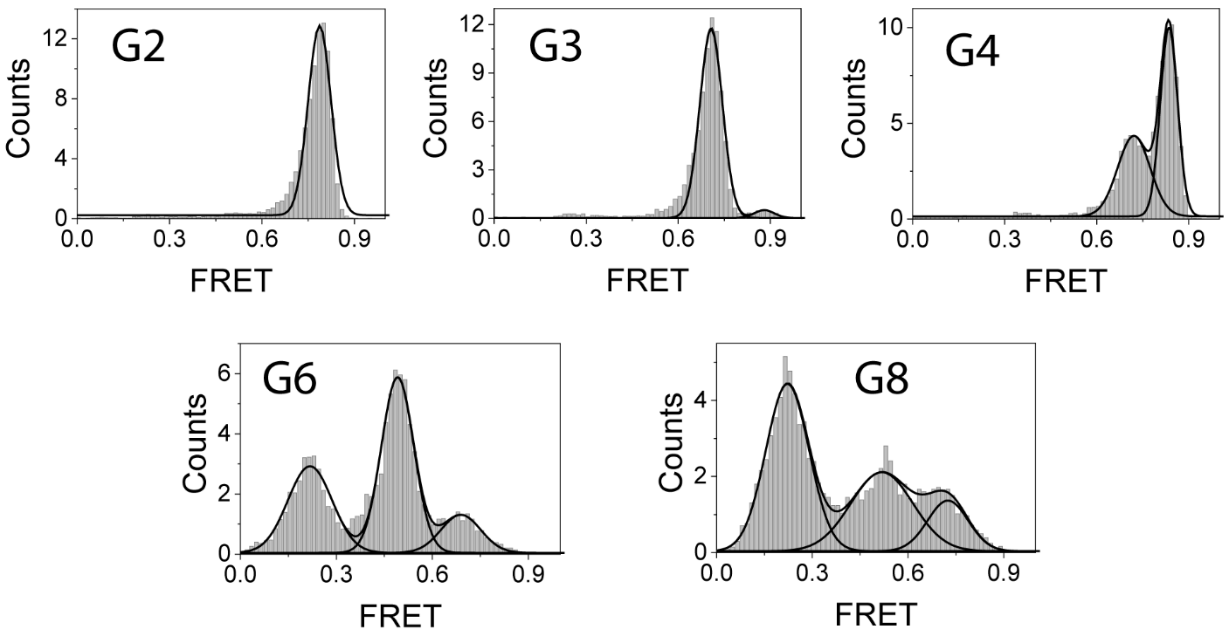

Figure S7: FRET histogram of G2, G3, G4, G6 and G8 telomere overhang during vectorial folding (by Rep-X, 10 nM) at 50 μM ATP concentration. The FRET histogram looks identical to high ATP concentration (1 mM). All the vectorial folding histograms shown here are taken after 3 minutes of folding. In case of G6 and G8, the low FRET peak at ~0.2 is the unfolded population at 3 min. This is may be due to less efficient of unfolding of longer duplex at low ATP concentration.

Supplementary Figure 8

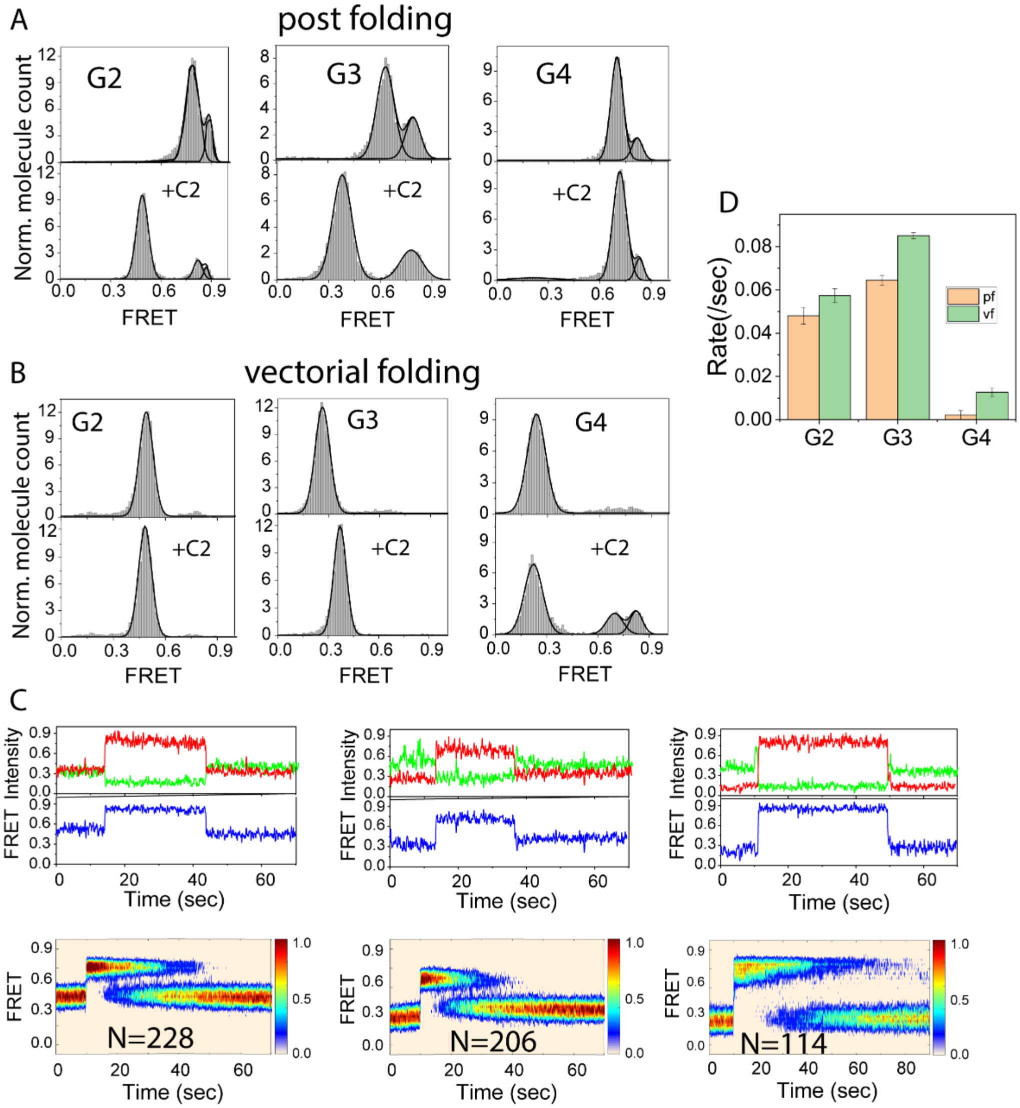

Figure S8: (A) FRET histograms of G2, G3 and G4 telomere overhang alone (top) and in the presence of two CCCTAA repeats (C2) (bottom) in post folding condition. **(B)** FRET histogram of G2, G3 and G4 telomeric duplex alone (top) and in the presence of two CCCTAA repeats (C2) in vectorial folding condition. In both cases, the C2 binding (250 nM) histograms shown here were obtained after 3 minutes of incubation at 100 mM KCl and 1 mM ATP. **(C)** Representative real-time smFRET traces showing unwinding of telomeric duplex (initial low FRET) and folding of telomere overhang (high FRET) followed by C2 binding on telomere overhang (again low FRET). Below, a heatmap generated by combining smFRET traces, synchronizing at the initial low to high FRET transition during vectorial folding and followed by C2 binding. The number of traces (N) used to generate the heatmap are mentioned accordingly. **(D)** C2 binding rate in post folding and vectorial folding of telomere overhangs obtained from the single-exponential fitting of C2 bound fraction (Figure 4B).

Supplementary Figure 9

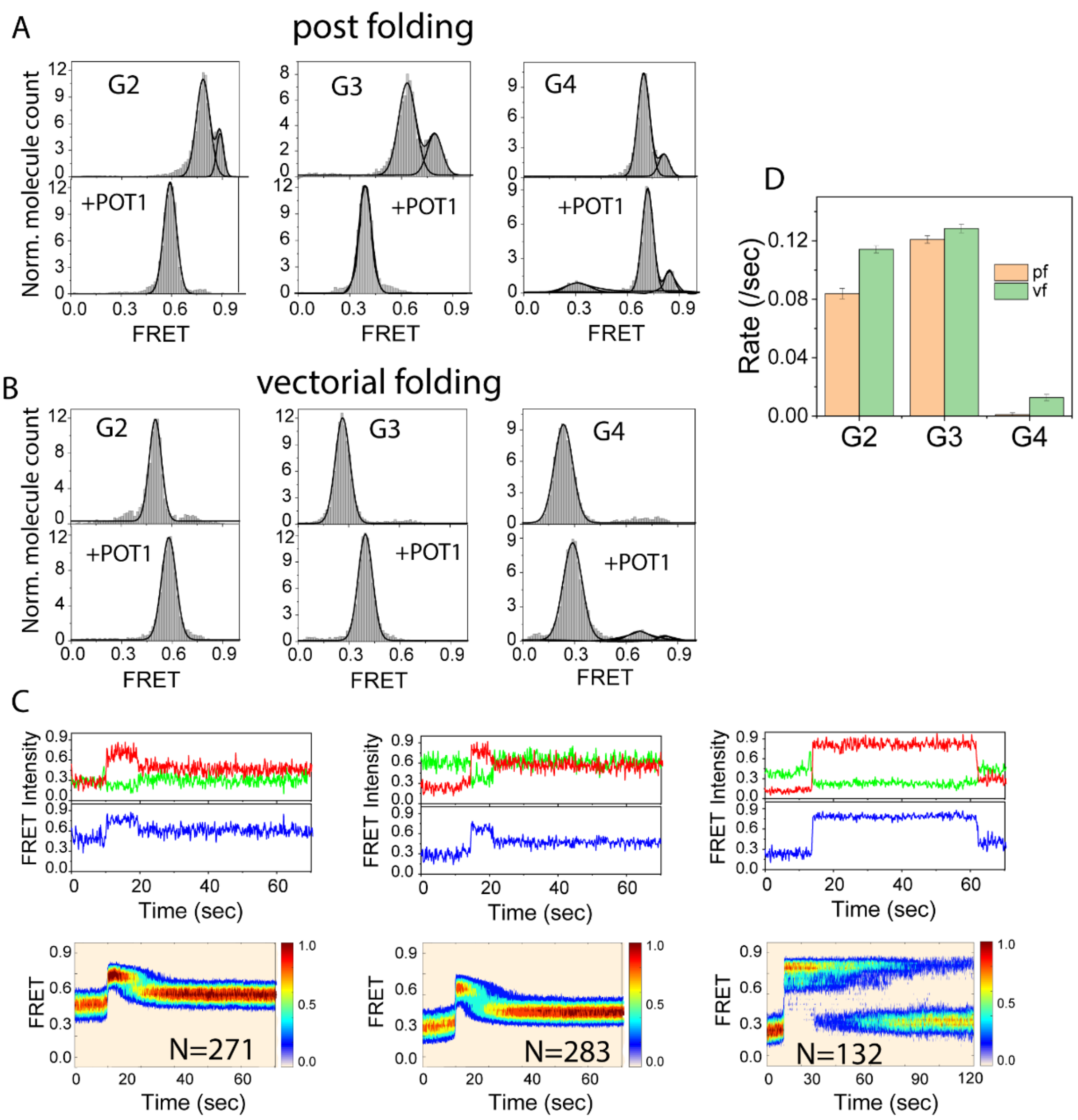

Figure S9: (A) FRET histograms of G2, G3 and G4 telomere overhang alone (top) and in the presence of two CCCTAA repeats (C2) (bottom) in post folding condition. **(B)** FRET histogram of G2, G3 and G4 telomeric duplex alone (top) and in the presence of two CCCTAA repeats (C2) in vectorial folding condition. In both cases, the C2 binding (250 nM) histograms shown here were obtained after 3 minutes of incubation at 100 mM KCl and 1 mM ATP. **(C)** Representative real-time smFRET traces showing unwinding of telomeric duplex (initial low FRET) and folding of telomere overhang (high FRET) followed by POT1 binding on telomere overhang (again low FRET). Below, a heatmap generated by combining smFRET traces, synchronizing at the initial low to high FRET transition during vectorial folding and followed by POT1 binding. The number of traces (N) used to generate the heatmap are mentioned accordingly. **(D)** POT1 binding rate in post folding and vectorial folding of telomere overhangs obtained from the single-exponential fitting of POT1 bound fraction (Figure 4D).

Supplementary Figure 10

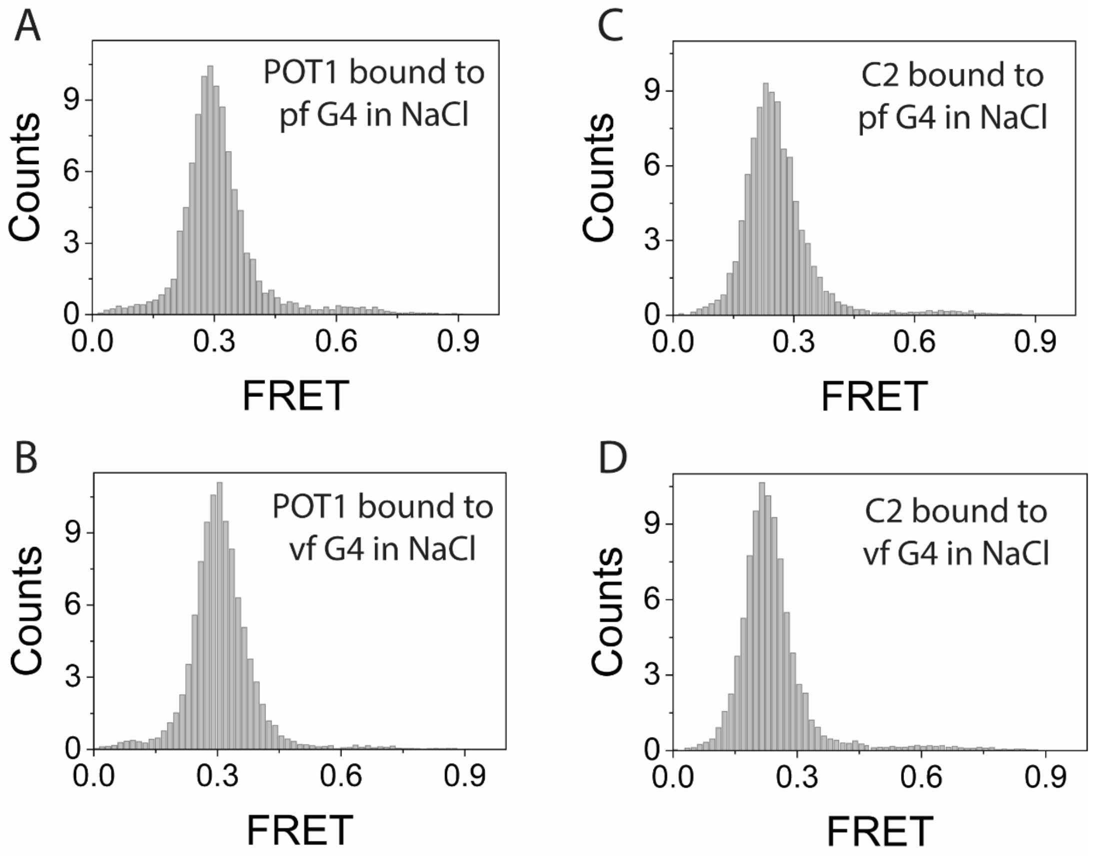

Figure S10: POT1 and C2 almost 100% accessible to both pf or vf G4 formed in NaCl. **(A & B)** POT1 bound histogram of pf and vf G4 in NaCl. **(C & D)** C2 bound histogram of pf and vf G4 in NaCl.

Supplementary Figure 11

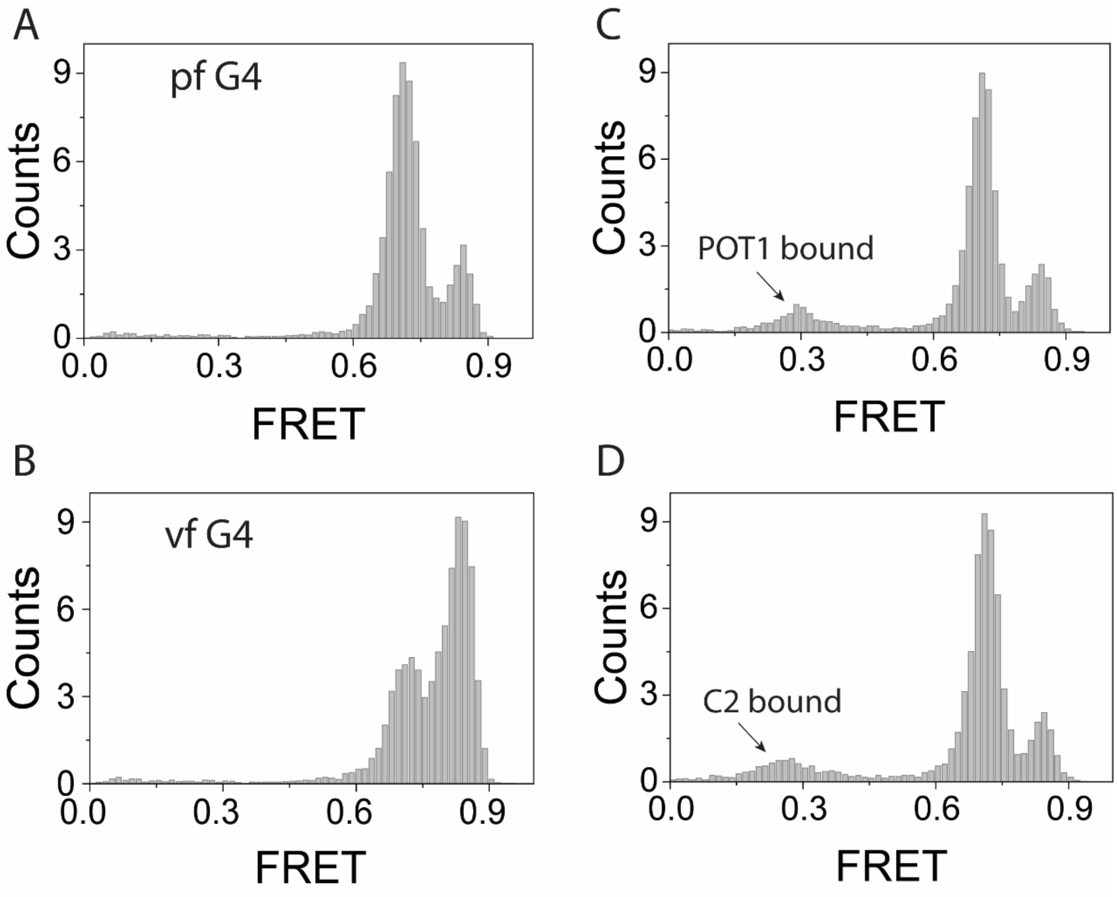

Figure S11: Rep-X neither alters conformational state nor influences C2 or POT1 binding to both pf or vf G4 in KCl. **(A)** Histogram of pf G4 in presence of Rep-X (10 nM) with ATP (1 mM). **(B)** Rep-X induced vf G4 histogram after 0.1% SDS wash.to ensure no contribution of Rep-X to vf G4 conformations. **(C & D)** POT1 and C2 bound histogram of pf G4 in presence of Rep-X with ATP. Less accessible (<10%) of POT1 and C2 to pf G4 in presence of Rep-X with ATP indicate that Rep-X doesn't influence for high accessible of POT1 and C2 to vf G4.

Supplementary Figure 12

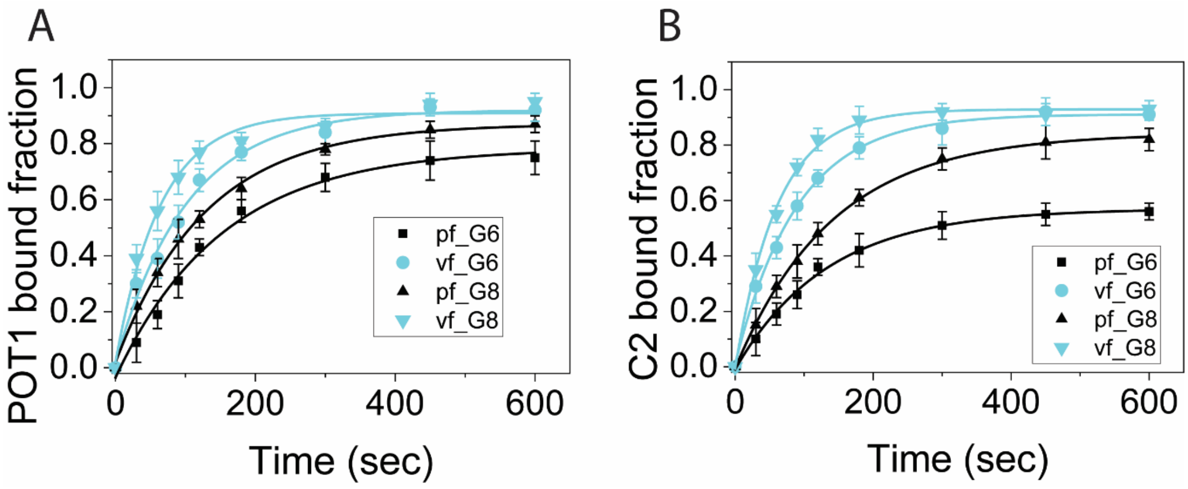

Figure S12: POT1 and C2 binding to pf or vf (using Rep-X at high ATP concentration) of G6 and G8 constructs in 100 mM KCl salt condition. Single-exponential fitting of POT1 (500 nM) bound fraction **(A)** or C2 (250 nM) bound fraction **(B)** to pf or vf of G6 and G8 constructs.

Supplementary Figure 13

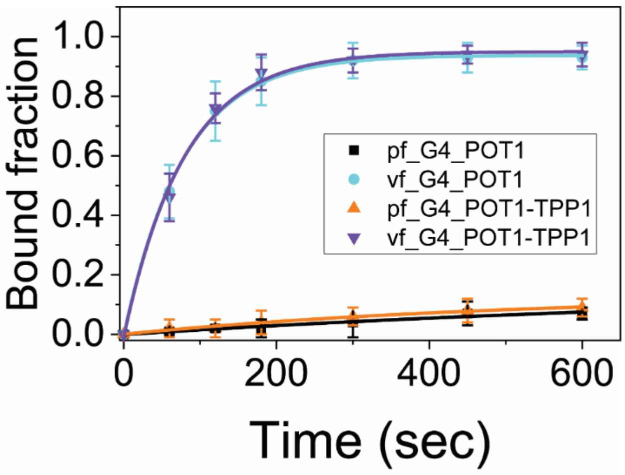

Figure S13: The POT1 binding accessibility does not change in the presence of the partner protein, TPP1 to both pf and vf G4 in KCl. Single-exponential fitting of POT1 bound fraction to both pf or vf G4 in presence or absence of TPP1.

Supplementary Figure 14

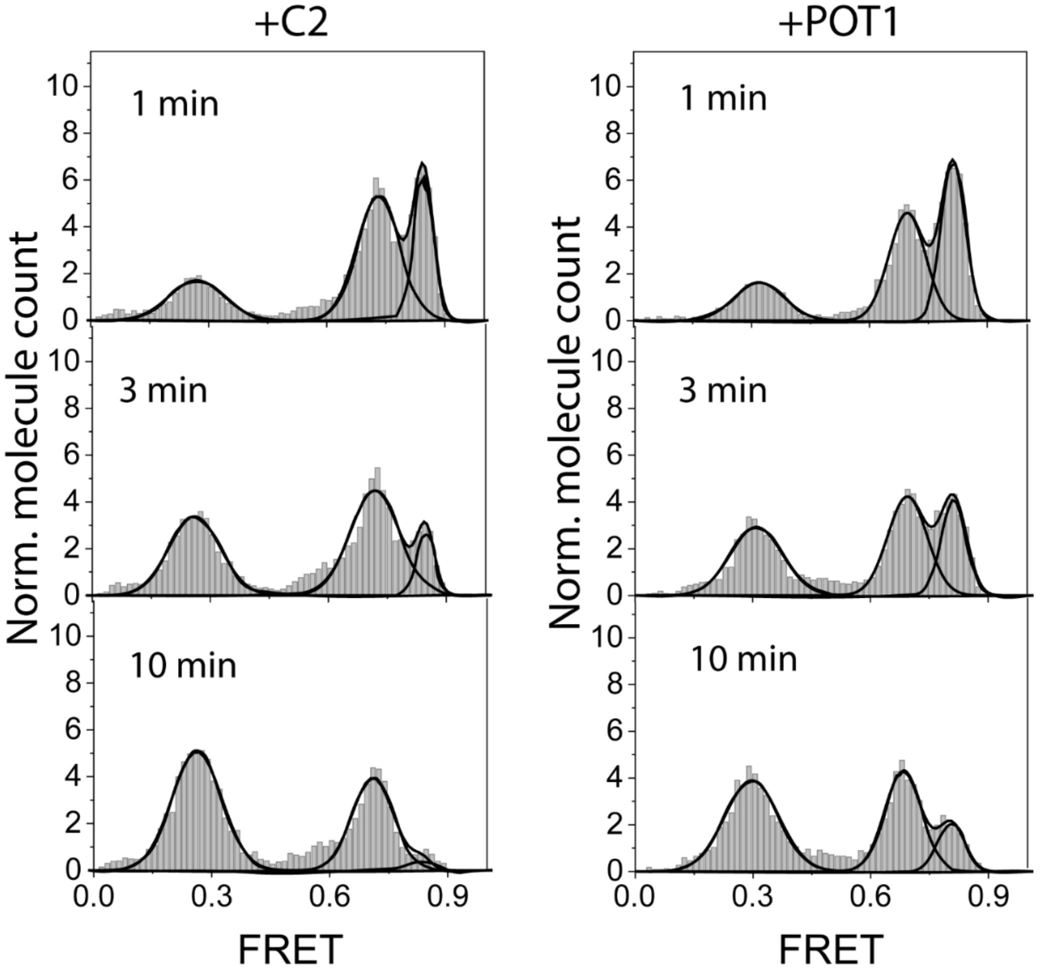

Figure S14: The accessibility test of C2 and POT1 binding to pf G4 in 100 mM LiCl to buffer exchange in 100 mM KCl. FRET histograms with time of C2 (left side) and POT1 binding (right side) during buffer exchange from LiCl to KCl.

Supplementary Figure 15

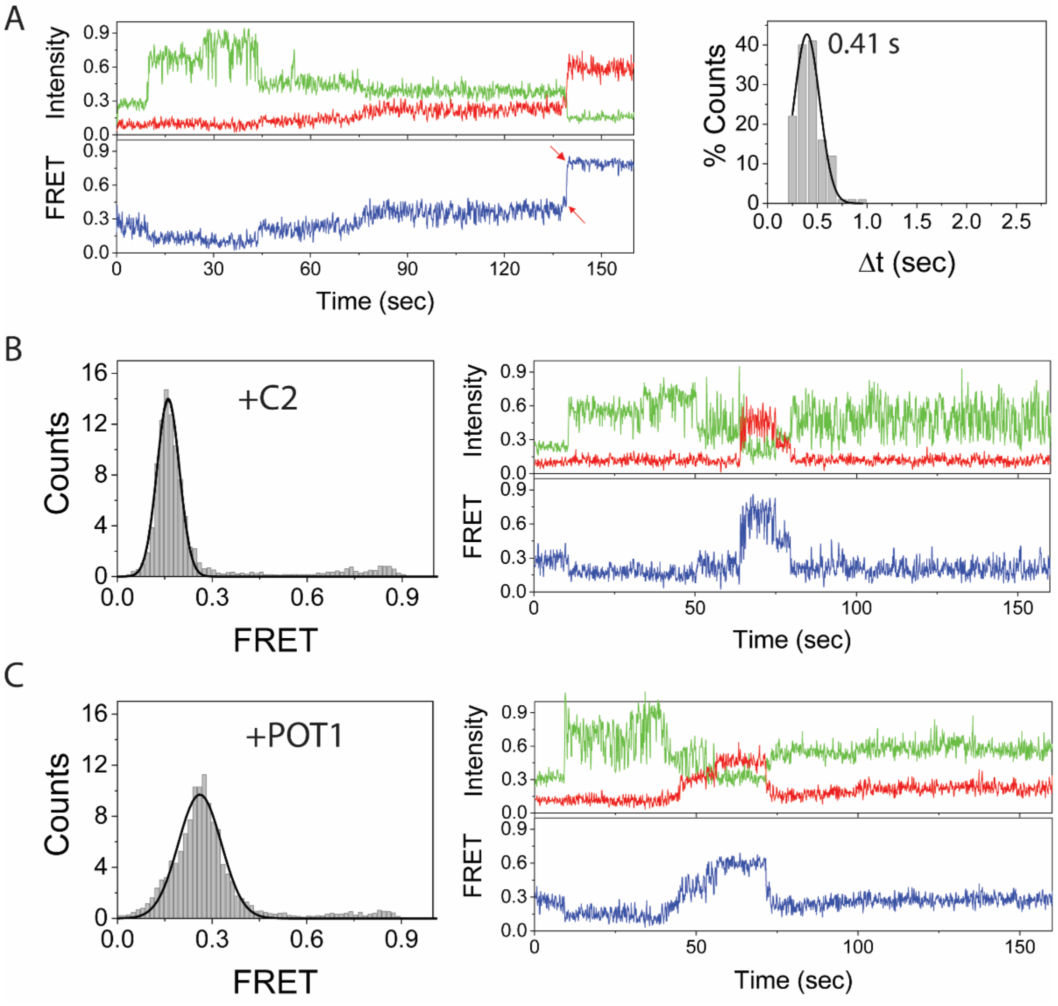

Figure S15: λ-exonuclease mediated vectorial folding and accessibility. **(A)** Real-time smFRET traces of λ-exonuclease mediated G4 vectorial folding. Besides, the Gaussian fit of the folding dwell time (~0.41 s) from the unfolded to folded state (red arrow indicated in flow traces). **(B & C)** FRET histogram of exonuclease mediated vectorial folding followed by C2 **(B)** or POT1 **(C)** binding. Real time C2 or POT1 binding smFRET traces during the exonuclease mediated vectorial folding.

Supplementary Figure 16

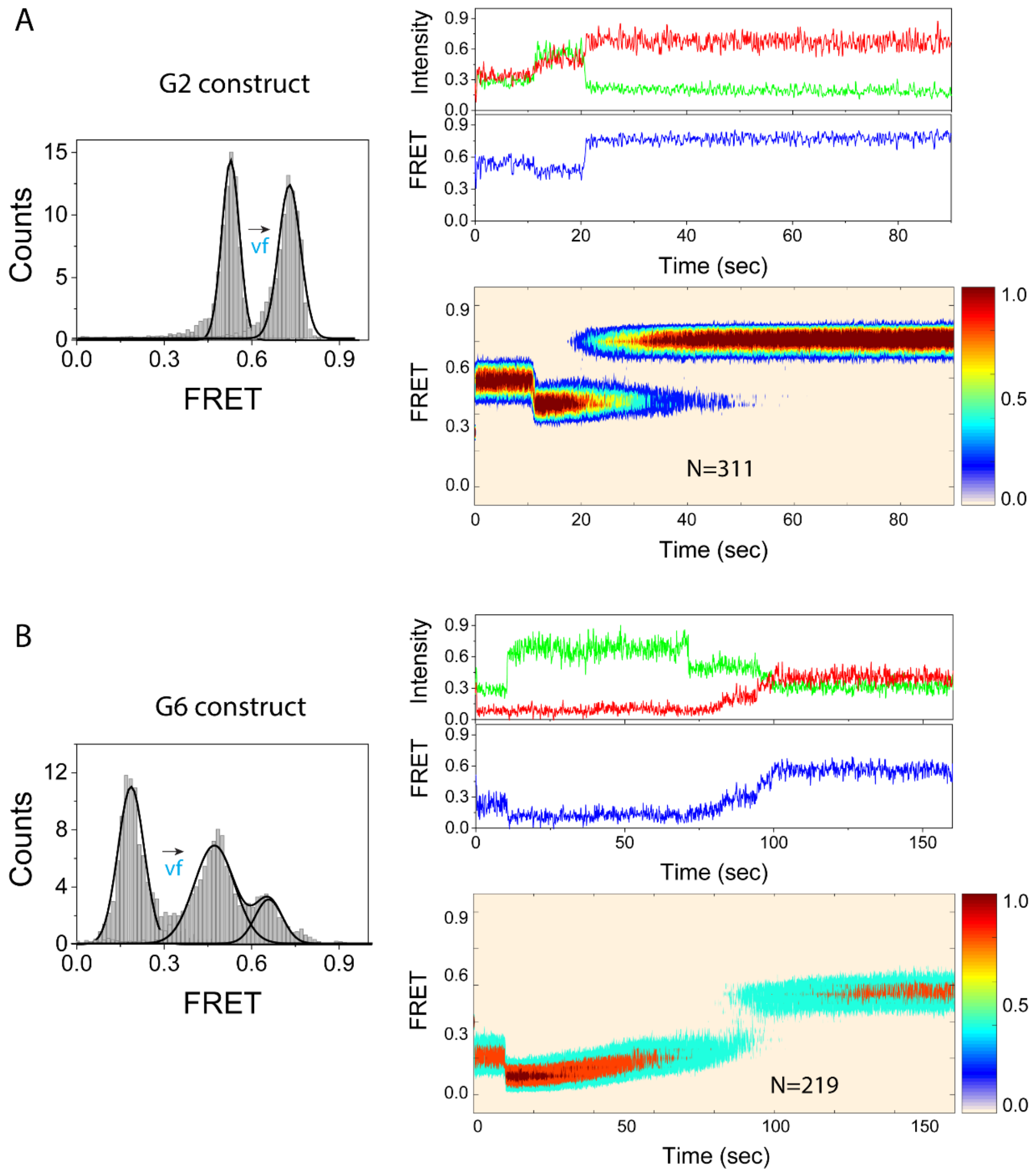

Figure S16: λ-exonuclease mediated vectorial folding of G2 **(A)** and G6 **(B)** constructs. **(A)** FRET histogram of G2 duplex (left side) and vectorial folding of G2 overhang (right side). Beside, real-time smFRET trace of λ-exonuclease mediated G2 vectorial folding (top) and the heatmap (bottom) generated by combining of smFRET traces (no synchronization). **(B)** FRET histogram of G6 duplex (left side) and vectorial folding of G6 overhang (right side). Beside, real-time smFRET trace of λ-exonuclease mediated G6 vectorial folding (top) and the heatmap (bottom) generated by combining of smFRET traces (no synchronization).
